# Mental health outcomes relating to climate-sensitive extreme weather events: A scoping review of measures of mental health impact

**DOI:** 10.1016/j.joclim.2026.100743

**Published:** 2026

**Authors:** Denise Thomson, Meghan Sebastianski, Katie O’Hearn, Megan Nuspl, Sol Bobier, Sami Aftab Abdul, Matt Pearce, Vijendra Ingole, Myer Glickman, Richard J Webster

**Affiliations:** a University of Alberta, Edmonton, Canada; b CHEO Research Institute, Ottawa, Canada; c Cochrane Planetary Health Thematic Group, London, United Kingdom; d Climate and Health Team, Office for National Statistics (ONS), Newport, United Kingdom; e Telfer School of Management, University of Ottawa, Ottawa, Canada

**Keywords:** Extreme weather events, Climate change, Mental health, Mental health impact, Mental health outcomes, Official statistics

## Abstract

**Introduction:**

Climate change is contributing to an increase in frequency and severity of extreme weather events (EWEs), which have a sizable impact on mental health and wellbeing. We sought to comprehensively synthesize measures of mental health impact used in the literature following EWEs.

**Materials and methods:**

This scoping review searched eight databases from 2017 to September 2024 to identify primary research studies that measured and reported mental health outcomes following EWEs. We categorized findings to describe a) which EWEs were most heavily studied, b) which mental health outcomes appeared more or less frequently, c) which measures of mental health impact were employed, and d) evidence gaps.

**Results:**

We screened 3,689 studies and included 154 studies from 29 countries, 15 (51.7%) of which were low- and middle-income countries. Studies reporting mental health outcomes were most commonly investigating the impact of 11 types of EWEs. We identified 188 mental health outcomes and 130 measures of mental health impact. Mental health outcomes were most commonly evaluated in studies investigating the impact of floods (n = 41), extreme heat/heatwaves (n = 32) and wildfires (n = 28). The most frequently evaluated mental health outcomes were post-traumatic stress disorder (n = 62), depression (n = 39) and anxiety (n = 27). The most commonly used measure of mental health impact was International Classification of Diseases (ICD-10) codes.

**Conclusion:**

This scoping review provides a global overview of current reporting practices and supports the need for standardization to enable global comparisons and decision-making.

## 1 Introduction

Evidence is accumulating that climate change is increasing the intensity, frequency, duration, and seasonality of extreme weather events (EWEs) such as floods, extreme heat/heatwaves and wildfires [1]. These events have a range of adverse impacts on global mental health and wellbeing, both directly, e.g. exposure to flooding or wildfires, need to seek medical attention after experiencing EWEs and/or indirectly, e.g. grief for the loss of traditional ways of life, undernutrition or displacement following drought or flooding [2]. EWEs can impact mental health through many different pathways, such as direct heat exposure, loss of access to green and blue spaces, disruptions to normal behavior patterns, damage to homes, and financial impacts [2,3]. Further, there is a real risk that climate change may erode hope, which must be nurtured in order to support humanity’s ability to meet this unprecedented challenge [4].

Previous evidence syntheses in this area have focused on assessing the impacts of climate change on mental health, often focusing on one specific EWE such as flooding or drought, or on identifying gaps in research [3,5,6]. However, a comprehensive overview of how we can measure and monitor the mental health impact of different EWEs is lacking. Given the breadth of mental health outcomes that may be impacted by EWEs and the complex pathways through which EWEs exert these impacts (e.g. direct and indirect, short-term and long-term, single and repeated exposures), different approaches to measurement are required for proper evaluation and monitoring. This is essential to provide researchers and policymakers with high-quality, accessible and reliable evidence and data to support the development, implementation and sustainment of adequate policy responses [7].

Recognizing the need to support decision-making for climate adaptation in health systems and better understanding of health outcomes, the UK Office for National Statistics (ONS), funded by Wellcome and in collaboration with the African Institute for Mathematical Sciences (AIMS) Rwanda, the Regional Institute for Population Studies (RIPS) at the University of Ghana, the UK Health Security Agency (UKHSA), and the Cochrane Planetary Health Thematic Group (hosted at University of Alberta) has developed the Standards for Official Statistics on Climate-Health Interactions (SOSCHI) project [8]. The goal of SOSCHI is to develop a transparent, globally generalizable, and spatially scalable (from local to national) framework and technical platform for official statistics on climate and health. Substantial foundational work to understand current practice is required to achieve this goal. As a first step, this scoping review aimed to synthesize the existing approaches to measuring the mental health impact of EWEs.

Our research question was: “What mental health impacts from extreme weather events due to climate change are being surveyed or reported in the literature and how are they being measured”. To answer this question, we conducted a scoping review led by the Cochrane Planetary Health Thematic Group (CPHTG), to map the currently available literature. Specifically, we sought to identify: (1) What are the mental health outcomes that are directly relevant to climate-sensitive EWEs; (2) What measures of mental health impact are relevant to mental health outcomes that are directly influenced by EWEs.

## 2 Methods

We performed a scoping review following the framework of Arksey and O’Malley [9] as well as the PRISMA-ScR extension for scoping reviews [10]. The scoping review method supports exploratory work related to broad research questions and is therefore well-suited to the purpose of this study [9]. With previous work showing a large increase in the number of new publications on this topic appearing since 2017, the author team agreed that focusing on studies from 2017 onwards would provide sufficient material to reach saturation [6]. Due to operational requirements of the SOCHI project, climate-sensitive EWEs of interest were defined as low pressure systems (cyclone, hurricanes, tornadoes, typhoons), extreme heat (extreme heat and heat waves), extreme cold (extreme cold and cold spells), extreme precipitation (extreme rainfall and snowstorms), drought, floods, and wildfires. The protocol for this review was published on SOSCHI (Standards for Official Statistics on Climate Health Interactions) Zenodo open repository (https://zenodo.org/records/15374228) on May 13, 2025.

### 2.1 Search strategy and selection criteria

The search strategy for this scoping review (Supplementary Material 1) was developed by an information specialist in consultation with the author team. The search terms captured the concepts of the EWEs listed above, mental health outcomes, and measures of mental health impact. We searched the following platforms and databases from 2017 to September 2024: Ovid MEDLINE ALL, Ovid Embase Classic+Embase, Ovid APA PsycInfo, Clarivate Web of Science's Science Citation Index and Emerging Sources Citation Index, Elsevier Scopus, EBSCO CABI Global Health, and Epistemonikos. Except for Epistemonikos (searched on September 29, 2024), all databases were searched on September 28, 2024. No limits were placed in the search on geographical location, language, or publication type.

All records returned through the electronic database search were screened independently at the title and abstract level by two reviewers using Covidence (https://www.covidence.org/). The full inclusion and exclusion criteria can be found in Table 1. Any records deemed potentially relevant were retrieved for further examination. Full text documents were further subjected to independent screening by two reviewers, and any uncertain judgements or disagreements were resolved by consensus discussions. All primary studies included at this stage were retained for data extraction. Relevant scoping and systematic reviews were retained for hand screening of their included studies. At all stages of screening, we piloted a subset of 20–30 studies to ensure consistency between reviewers.

**Table 1:** Inclusion and exclusion criteria.

	Include	Exclude
**Study design**	Primary research studies (cohort, cross-sectional, case series (more than 10 patients). Include scoping and systematic reviews at secondary screening to screen included primary studies	Opinion, narratives, case study, letters to editor, laboratory studies
**Topic**	Climate change impacts	Climate change adaptation or resilience
**Population**	Human studies	Animal studies, plant/crop/grass studies
**Exposure**	Extreme weather due to climate change: Extreme temperatures: cold, heat, severe winter conditions such as snow/ice or frost/freeze. Storm: extra-tropical storms, tropical storms, convective storms – includes derecho, hail, lightning/thunderstorm, rain, tornado, sand/dust storm, storm/surge, wind, cyclone, typhoon Flood: coastal flood, riverine flood, flash flood, ice jam flood Avalanche: snow debris, mudflow Drought Wildfire: forest fire, land fire, brush fire, bush fire, pasture fires	Extreme events that are not climate related: Earthquake: ground movement, tsunami Dry mass movement: rockfall, landslide Volcanic activity: ash fall, lahar, pyroclastic flow, lava flow Epidemic: viral, bacterial, parasitic, fungal, prion Insect infestation: grasshopper, locust Animal accident Space weather: geomagnetic storm Human-made disasters: chemical spill, explosion, gas leak
**Measure of mental health impact**	Any: ICD codes Symptom scales, Screening tools Administrative data: hospital admissions, mortality, ER visits	

**Outcome**	Mental health disorders (anxiety, depression, PTSD, acute stress, suicidal behavior, intentional self-harm, mood disorder, dementia, Alzheimer disease, schizophrenia, schizotypal and delusional disorders, acute and transient psychotic disorders, psychosis, substance use and addiction)	Domestic abuse, general climate change anxiety, psychological and behavioral disorders associated with sexual development and orientation, disorders of psychological development

### 2.2 Data extraction

A data extraction template was developed in Excel (Microsoft) through consultation between the ONS and the CPHTG, and was piloted by the reviewers completing data extraction to ensure inter-reviewer consistency. Data was extracted by one reviewer and verified by a second. Any inconsistencies were noted and resolved by consensus discussions. Key information was extracted from each record, including authors or producing organization, year of publication, mental health outcomes presented and available details for any breakdown of data by demographic and social characteristics/intersectionality, and measures of mental health impact (e.g. International Classification of Diseases (ICD) codes, standardized questionnaires, rating scales).

### 2.3 Analysis of evidence

We categorized our findings to describe a) which of the included EWEs were the most heavily studied, b) which mental health outcomes appeared more or less frequently, c) which measures of mental health impact were employed, and d) where any evidence gaps existed. Countries were categorized as low- and middle-income countries (LMICs) using Wellcome’s list of LMICs, which is based on a classification updated every three years by the Organization for Economic Cooperation and Development (OECD) [11].

### 2.4 Measures of mental health impact and mental health outcomes

Due to the operational requirements of the SOCHI project, we focused on studies reporting direct mental health outcomes from EWEs (e.g. anxiety, depression, PTSD, acute stress, suicidal behavior, intentional self-harm, mood disorder, dementia, Alzheimer disease, schizophrenia, schizotypal and delusional disorders, acute and transient psychotic disorders, psychosis, substance use and addiction), and excluded studies reporting indirect outcomes (e.g. domestic abuse, general climate change anxiety, psychological and behavioral disorders associated with sexual development and orientation, disorders of psychological development). We sorted mental health outcomes into categories based on the groupings of diagnostic criteria and codes from the Diagnostic and Statistical Manual of Mental Disorders, Fifth Edition (DSM-5) and the International Classification of Diseases 10th and 11th revisions. For each mental health outcome, we categorized the measures of mental health impact used and how those data were collected at the study level. Measures of mental health impact included both primary sources such as symptom scales, screening tools, surveys and questionnaires, and secondary sources such as International Classification of Diseases (ICD) codes which are recorded mainly in administrative data (hospital admissions, mortality, emergency room (ER) visits).

## 3 Results

The database search returned 2,921 unique records. An additional 1,009 records were identified from handsearching relevant systematic reviews before duplicates were removed. After duplicate removal, a total of 3,689 records were screened with 154 primary studies included in this scoping review (see Supplementary Material 2 for list of included studies and their characteristics). Fig. 1 shows the flow of records through a PRISMA diagram. The 154 studies were conducted in 29 countries, 15 of which were LMIC (51·7%). Overall, 64 studies were conducted in LMICs, with a few studies including multiple countries: China (n = 21), India (n = 13), Philippines (n = 9), Bangladesh (n = 5), Indonesia (n = 5), Vietnam (n = 3), Botswana (n = 2), and 1 study each for Brazil, Burundi, Ethiopia, Ghana, Malaysia, Pakistan, South Africa and Thailand. Ninety-eight studies were conducted in high-income countries, again with a few studies including multiple countries: United States (n = 37), Australia (n = 20), Canada (n = 14), United Kingdom (n = 9), three studies each for Japan, South Korea and Spain, two studies each for Switzerland and Taiwan, and one study each for the Bahamas, Belgium, Finland, Greece and Sweden. Most studies (n = 31) were published in 2019, followed by 2021 (n = 27), 2018 (n = 25), 2017 (n = 23), 2020 (n = 17), 2022 (n = 15), 2023 (n = 11) and 2024 (n = 5).

**Fig. 1:**
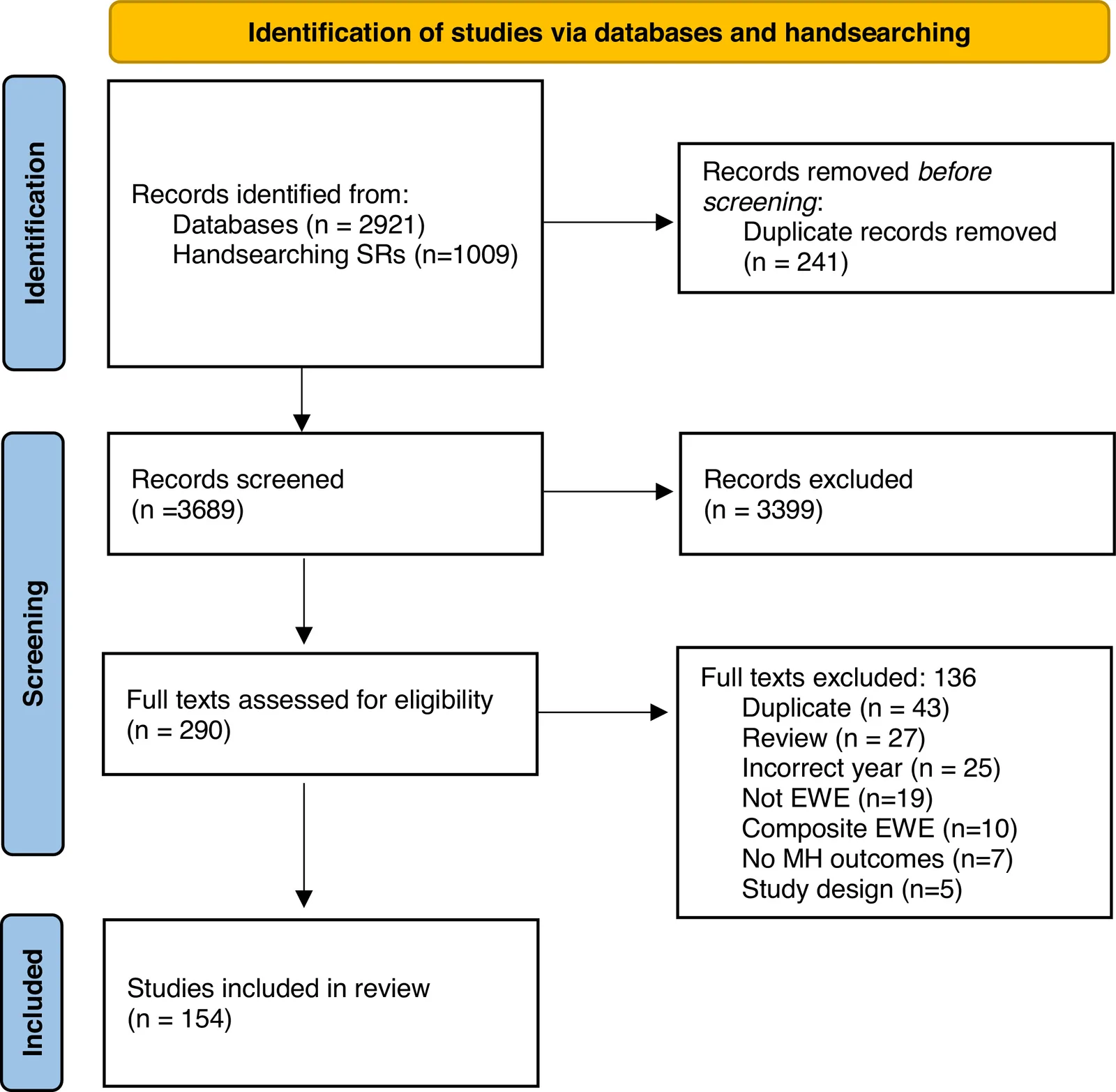
Study selection.

From the included studies, we identified 11 types of climate change-related EWEs for which the impacts on mental health were measured. Mental health outcomes were most often evaluated in studies investigating the impact of floods (41 studies), extreme heat / heat waves (32 studies), and wildfires (28 studies). Other types of EWEs studied in relation to mental health impacts were hurricanes (21 studies), droughts (12 studies), typhoons (9 studies), extreme cold / cold spells (6 studies), tornados (6 studies), cyclones (3 studies), extreme rainfalls (2 studies), and snowstorms (2 studies).

Overall, the studies evaluated 188 mental health outcomes which we classified into 17 DSM-5 categories (Fig. 2) (see Supplementary Material 3 for summary of mental health outcomes within each DSM-5 category). Outcomes of Trauma and Stressor-Related Disorders had the highest diversity of mental health outcomes (n = 43), followed by General Mental Health Outcomes (n = 21), and Substance-Related and Addictive Disorders (n = 21), while three categories had only one outcome (Bipolar, Neurodevelopmental, Obsessive-Compulsive and Related Disorders). Across these categories, the five most reported unique outcomes were post-traumatic stress disorder (PTSD) (n = 62), depression (n = 39), anxiety (n = 27), psychological distress (n = 12) and suicidal ideation (n = 12). The Sankey diagram in Fig. 3 shows the links between EWEs and mental health outcome categories evaluated. Trauma- and stressor-related disorders were the most frequently evaluated category (n = 142), followed by depressive (n = 69) and anxiety disorders (n = 56). Studies focused on floods and wildfires evaluated the greatest number of mental health outcomes at 138 and 101 respectively. The most common mental health outcomes per EWE are reported in Table 2. A complete disaggregated list of mental health outcomes by EWE is available in Supplementary Material 4. Fig. 4 shows the relationship between EWE and mental health outcomes, with bubble size indicating the frequency of reported outcomes and color intensity representing the number of unique measures of mental health impact used. Studies of floods and low-pressure systems most frequently evaluated trauma- and stressor-related disorders, while other outcomes like depressive and anxiety disorders were evaluated in studies focused across multiple EWE types. A gap analysis revealed that many of the mental health categories were not measured for all EWEs; 36% (33/91) of EWEs by mental health categories had a gap.

**Fig. 2:**
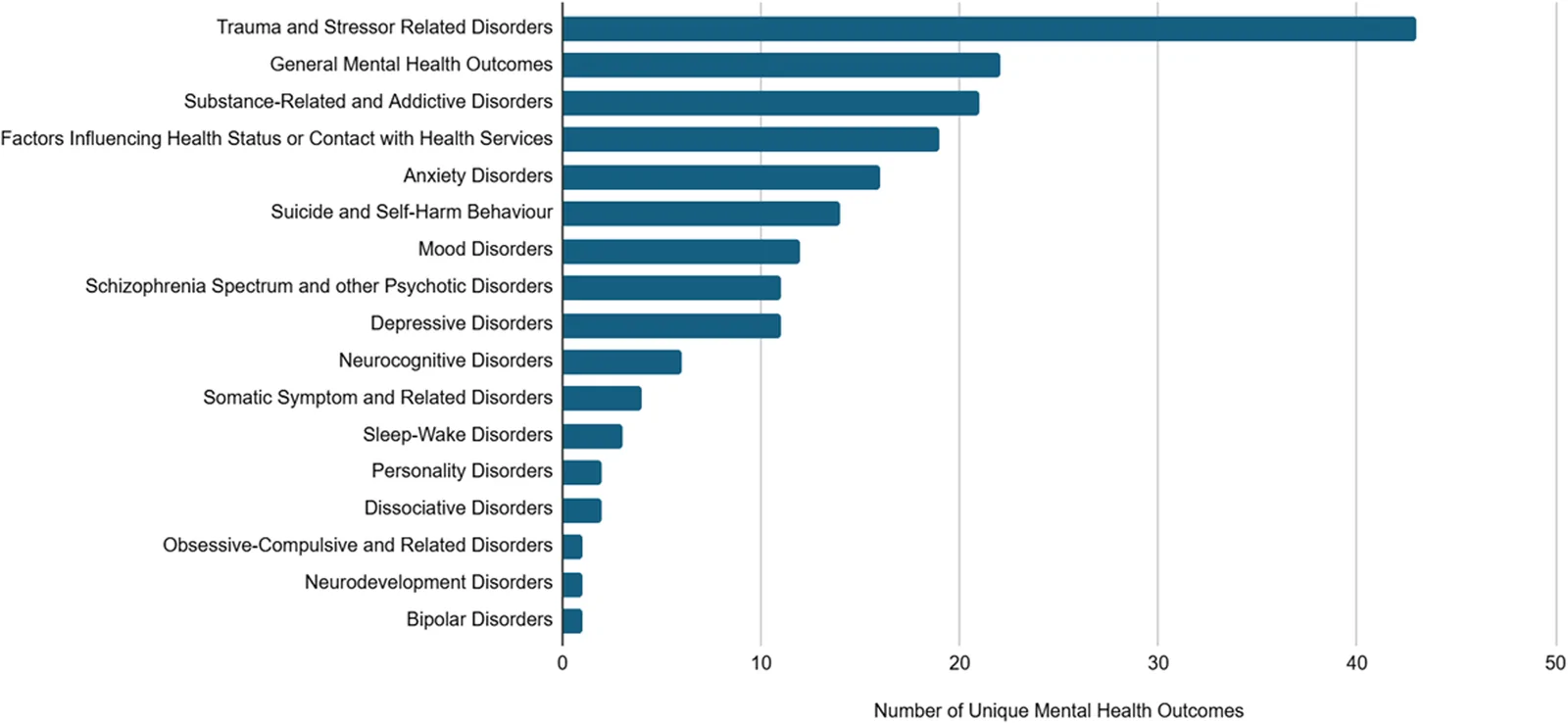
Frequency of reporting for each mental health outcome sorted into categories based on the groupings of diagnostic criteria and codes from the Diagnostic and Statistical Manual of Mental Disorders, Fifth Edition (DSM-5) and the International Classification of Diseases 10th and 11th revision.

**Fig. 3:**
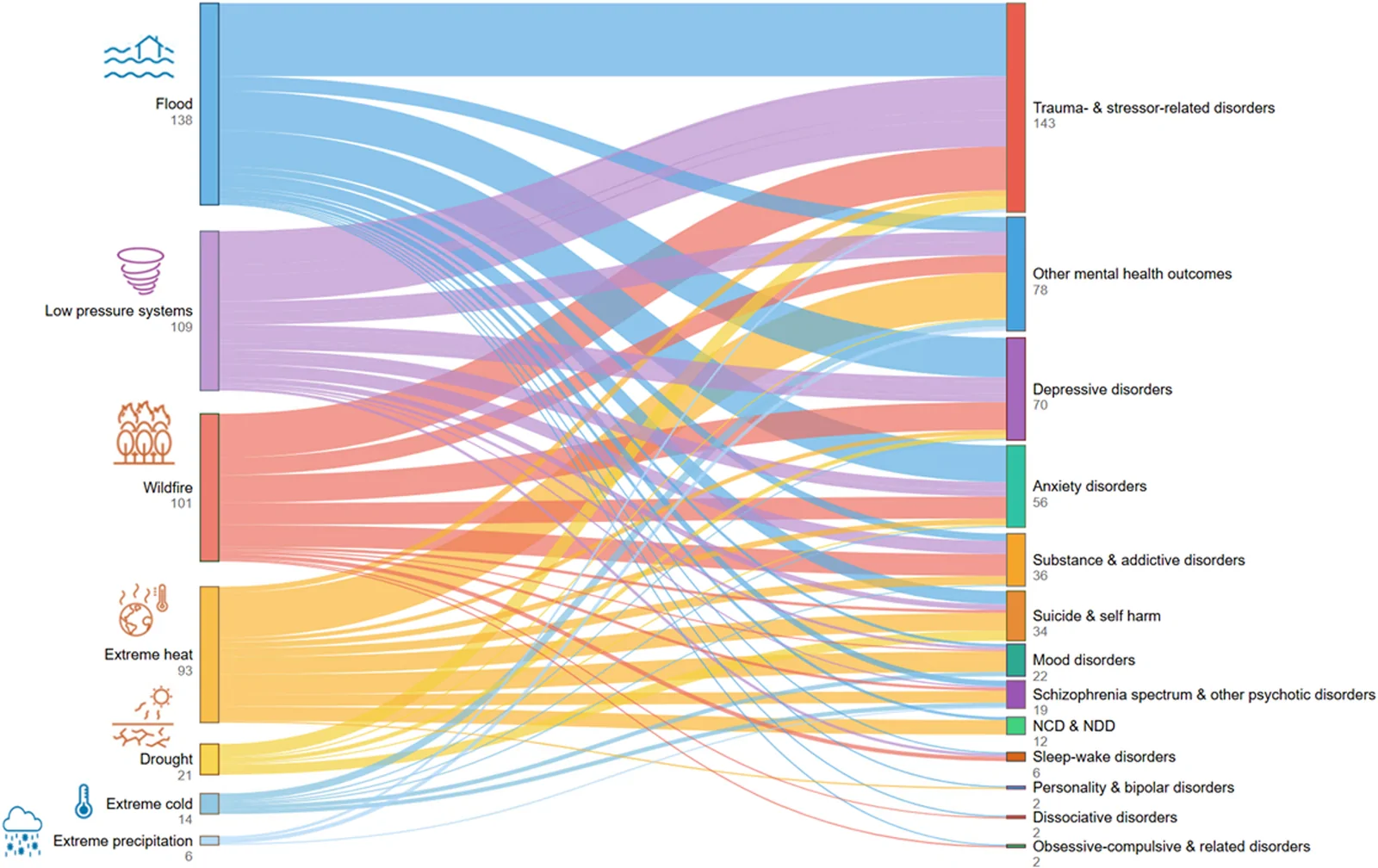
Frequency of mental health outcomes by EWE. EWEs definitions: Low pressure systems = Cyclone, hurricane, tornado, and typhoon; Extreme heat =Extreme heat/Heat waves; Extreme cold=Extreme cold/cold spells; Extreme precipitation = extreme rainfall, snowstorms Mental health definitions: NCD & NDD=Neurocognitive & neurodevelopmental disorders. Other mental health outcomes= general mental health outcomes, somatic symptom and related disorders, factors influencing health status or contact with health services.

**Table 2:** Summary of EWEs and frequency of most common mental health outcomes.

EWE (# of studies)	Countries (frequency)	# of unique MH outcomes	Three most common MH outcomes (frequency)
Cyclone (n = 3)	Bangladesh (2), India	5	1. Depression (3) 2. Anxiety (2) 3. (tied) Psychological distress; Posttraumatic Stress Disorder (PTSD); Suicidal ideation (1 each)
Drought (n = 12)	Australia (4), Bangladesh, Botswana (2), Ethiopia, India (3), USA	14	1. Depression (3) 2. (tied) Psychological distress; Suicide deaths; Suicide ideation; Trauma symptoms (2 each)
Flood (n = 41)	Australia (2), Bangladesh (2), Burundi, Canada, China (7), Ghana, India (9), Malaysia (1), Indonesia (5), Pakistan, Spain, Thailand, UK (7), USA (2)	64	1. Posttraumatic Stress Disorder (PTSD) (29) 2. Depression (22) 3. Anxiety (17)
Extreme Cold / Cold spells (n = 6)	China (4), Sweden, USA	13	1. Mental health ED visits (1) 2. All other outcomes occurred only once
Extreme Heat / Heat waves (n = 32)	Australia (3), Belgium, Brazil, Canada, China (7), Finland, India (2), Japan (3), Philippines, South Africa, South Korea (2), Spain (2), Sweden, Switzerland, Taiwan, UK USA (8), Vietnam (3)	54	1. Mental disorders admissions (8) 2. (tied) Mental health ED visits; Suicide deaths (6 each)
Extreme Rainfall (n = 2)	China (2)	4	1. (tied) Posttraumatic Stress Disorder (PTSD); Resilience; Rumination; Schizophrenia hospitalizations (1each)
Hurricane (n = 21)	Bahamas, Puerto Rico, USA (19)	36	1. Posttraumatic Stress Disorder (PTSD) (8) 2. Depression (5) 3. Anxiety (3)
Snowstorm (n = 2)	UK, USA	2	1. (tied) Emotional upheaval; Posttraumatic Stress Disorder (PTSD) (1 each)
Tornado (n = 6)^	China (5), USA	9	1. Posttraumatic Stress Disorder (PTSD) (4) 2. Mindfulness (3) 3. (tied) Burnout; Depression (2 each)
Typhoon (n = 9)	Philippines (8), Taiwan	19	1. Posttraumatic Stress Disorder (PTSD) (5) 2. (tied) Acute stress disorder; Depression, anxiety and stress; Posttraumatic cognitions; Psychological distress (2 each)
Wildfire (n = 28)	Australia (11), Canada (12), Greece, USA (4)	48	1. Posttraumatic Stress Disorder (PTSD) (17) 2. Depression (10) 3. (tied) Anxiety; Major Depressive Disorder (6 each)

^5 of the 6 studies used the same baseline population data; EWE – Extreme weather event, MH – Mental health.

**Fig. 4:**
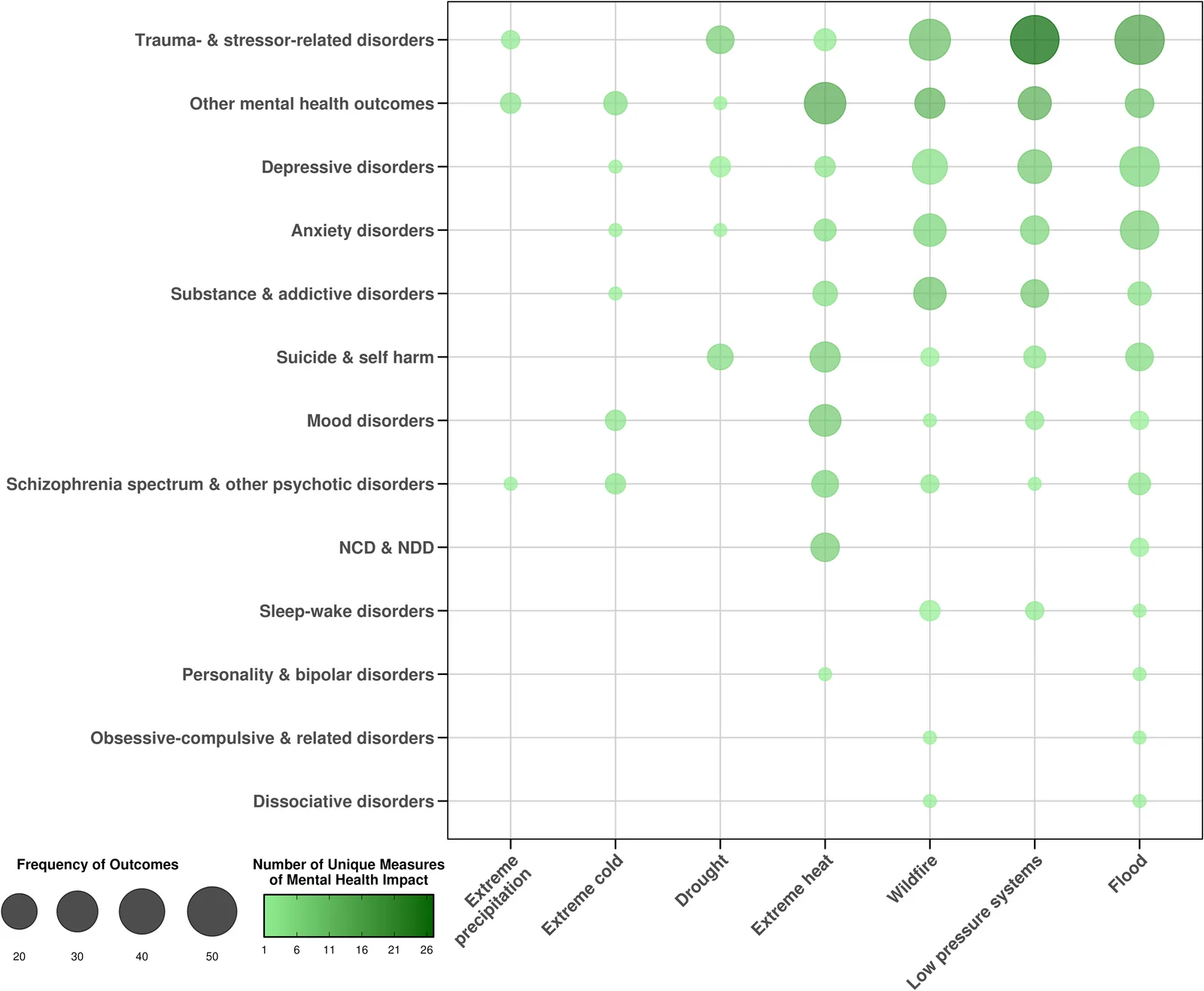
Gap analysis of reported mental health outcomes and EWEs EWEs definitions: Low pressure systems = Cyclone, hurricane, tornado, and typhoon; Extreme heat =Extreme heat/Heat waves; Extreme cold=Extreme cold/cold spells; Extreme precipitation = extreme rainfall, snowstorms. Mental health definitions: NCD & NDD=Neurocognitive & neurodevelopmental disorders. Other mental health outcomes= general mental health outcomes, somatic symptom and related disorders, factors influencing health status or contact with health services.

We identified a total of 130 measures of mental health impact. The most commonly used measure was ICD-10 codes with a frequency of 83 followed by the Patient Health Questionnaire - 9 (PHQ-9) (n = 25), ICD-9 (n = 21), Depression Anxiety and Stress Scales (DASS-21) (n = 20), and the Post-traumatic Stress Disorder Checklist for DSM-5 (PCL-5) (n = 17). ICD codes from the 8, 9 and 10th revisions were reported, with 67 mental health outcomes being reported by ICD codes. A summary of the five most common mental health outcomes and the type and frequency of the most used measures for each outcome is presented in Table 3. The number of measures used for each outcome varied widely. The mental health outcome of “PTSD,” categorized under Trauma and Stressor-Related Disorders, and the outcome “depression,” categorized under Depressive Disorders, had 22 and 15 measures respectively. Of the remaining outcome measures, 84% (157/188) had only one associated measure. The frequency distribution of unique measures across mental health categories by EWEs is summarized in Fig. 4. A full list of mental health outcomes and measures (divided into primary and secondary sources) can be found in Supplementary Material 3.

**Table 3:** Summary of most common mental health outcomes and type and frequency of measures of mental health impact.

Five Most Common MH Outcomes	Number of unique measures for that outcome	Three most common measures for that outcome (frequency)
1. Posttraumatic Stress Disorder	22	1. PTSD Checklist for DSM5 (PCL 5) (16) 2. PTSD Checklist (PCL−6) (8) 3. PTSD Checklist - Specific (PCL-S) (7)
2. Depression	15	1. Patient Health Questionnaire - 9 (PHQ-9) (15) 2. Patient Health Questionnaire – 2 (PHQ-2) (6) 3. Depression Anxiety and Stress Scales (DASS-21) (4)
3. Anxiety	13	1. (tied) Generalized Anxiety Disorder – 7 (GAD-7); Generalized Anxiety Disorder – 2 (GAD-2) (6 each) 2. Depression Anxiety and Stress Scales (DASS-21) (4)
4. Psychological distress	4	1. Kessler Psychological Distress Scale (K6) (7) 2. Kessler Psychological Distress Scale (K10) (3) 3. (tied) Brief symptom rating scale (BSRS-5); General Health Questionnaire Twelve Plus R (GHQ-12-Plus-R) - Items 1–12 (1 each)
5. Suicidal Ideation	10	1. Patient Health Questionnaire – 9 (PHQ-9) final item (2) 2. Interview questions (2) 3. All other tools had a frequency of 1

MH – Mental Health.

## 4 Discussion

The main purpose of this scoping review was to map the currently available measures of mental health impact relevant to mental health outcomes that are directly influenced by EWEs. In turn, this supports the development of transparent and well-designed official statistics on climate change and health [8]. Mental health outcomes and measures found were abundant and diverse. We identified 188 unique mental health outcomes, of which PTSD, depression and anxiety were most commonly evaluated, and 130 unique measures of mental health impact. Currently the SOSCHI framework has one indicator on mental health: suicides attributable to extreme heat [12]. These results highlight that suicides are a small proportion of the mental health outcomes associated with EWEs. This review can help to inform the development and prioritisation of any future additional SOSCHI indicators. Flooding, extreme heat/heat waves and wildfires were the most frequently studied EWEs. LMICs were well represented, comprising just over half of the 29 countries.

### 4.1 Heterogeneity in measures of mental health impact

Not surprisingly, there is a high heterogeneity in the measures of mental health impact used, highlighting an important opportunity for standardization. The variety of available measures has been previously identified in many contexts as a problem in determining the prevalence of mental health outcomes [[13], [14], [15]]. For example, in this review depression and PTSD were studied using 15 and 22 unique measures respectively, the majority of which were patient-reported questionnaires or screening tools. This heterogeneity makes it challenging to accurately quantify the breadth of the mental health burden associated with EWEs.

### 4.2 Limitations of identified measures of mental health impact

The high use of questionnaires/surveys can pose challenges for generating official statistics reports as it presents concerns about instrument validity and the representativeness (and generalizability) of the sample [16]. This is particularly noteworthy, as mental health outcomes are very difficult to measure without primary sources such as questionnaires and surveys. This was demonstrated in our results where most of the measures of mental health impact used were primary sources. However, for regular reporting of official statistics wide-scale population surveys are unrealistic. Accordingly, administrative data must be used for official statistics, but this practice also poses its own challenges with respect to being representative of the true impacts.

It is also worth noting that some measures identified in this review, such as the Impact of Event Scale – Revised (IES-R) and PTSD Checklist for DSM-5 (PCL-5) tools, are intended to *screen* for symptoms of a mental health outcome (e.g., PTSD) but additional assessment is required to finalize the diagnosis [17]. Thus, care must be taken when interpreting reports as to whether the measure is being used to determine the prevalence of symptoms, or the prevalence of a diagnosed mental health condition. Establishing core outcomes and metrics for EWE impacts on mental health that support evidence synthesis and official statistics would improve consistency, support comparability and facilitate meta-analysis.

### 4.3 Use of administrative health data as a measure of mental health impact

ICD codes were the most frequently used measure of mental health impact following EWEs. ICD codes are typically used in healthcare records and also in death certification. They differ in nature from primary sources such as rating scales in that ICD codes represent the endpoint of a diagnostic and classification process, to which one or more primary sources may have provided input along with clinical expertise. This scoping review identified the mental health ICD codes associated with EWEs, used to quantify 67 mental health outcomes. The ICD codes identified in this review provide decision makers with a comprehensive list of codes that can be used to track and report on mental health outcomes associated with EWEs. The frequent use of ICD codes supports decision-making needs, as they are easily operationalizable for national official statistics, provide a standardized way of reporting outcomes, and allow for comparisons over time.

### 4.4 Limitations of administrative health data as a measure of mental health impact

However, ICD codes are not a panacea for providing mental health indicators relating to extreme weather events. Caution is needed in interpreting the most frequently reported ICD code categories as the most important mental health outcomes, as ease of measurement might drive their ubiquity, rather than actual mental health impact. It should be noted that the presence of ICD codes representing a long-term mental health condition or intellectual disability in a healthcare record does not necessarily suggest a causal relationship between the EWE and that condition but may rather reflect the vulnerability of individuals with a pre-existing diagnosis to the effects of the EWE exposure.

The accuracy of ICD codes is dependent on the expertise of the individual assigning the code, while diagnostic and classification practice may vary between countries and variability in coding has been observed across settings [18,19]. For example, stigma regarding mental health is variable by culture which may deter individuals from seeking care or reporting mental health concerns, leading to underreporting in certain regions [20]. There is variability in classification definitions across ICD versions and health systems are slow to adopt newer versions. Finally, national-level ICD coded data is currently limited or unavailable in some countries [21]. Despite these challenges, ICD codes can be an effective tool to report and track health outcomes, particularly when paired with efforts to address some of the above limitations, such as policy and training to support more consistent implementation internationally.

### 4.5 Study design of included studies and mental health impact of EWEs

Mental health outcomes related to specific EWEs also varied in abundance and diversity. All 11 EWEs identified evaluated a range of mental health outcomes, ranging in number from 64 mental health outcomes related to flooding to two mental health outcomes for snowstorms. The relationship between EWEs and multiple mental health outcomes from research spanning 29 unique countries is consistent with findings from numerous other studies showing that climate change is a major threat to global mental health [6,22]. However, based on the study design of included studies, we cannot definitively say that EWEs caused worse mental health outcomes. Our objective was to report the frequency with which mental health outcomes were evaluated, in the context of which EWEs, and the measures of mental health impact that were utilized and not to evaluate cause and effect. The majority of included studies were cross-sectional studies that looked at acute rather than long-term impacts This may have led to an underestimate of the impact of EWEs on mental health, as the impact of chronic exposures may not be adequately captured.

### 4.6 Existing gaps

The extent of the impact of EWEs on mental health remains understudied. Our scoping review, in common with other recent work, found that there are still significant research gaps in the relationship of climate-sensitive EWEs and mental health outcomes [22]. However, it is challenging to interpret what these gaps mean, as absence of evidence cannot be assumed to represent an evidence of absence. More broadly, much work on the effectiveness of climate-mental health adaptations and projected future risks for mental health is needed, accompanying the development and dissemination of indicators to support decision-making [6]. This was highlighted in the recent UK Health Security Agency climate change and mental health report which called for additional research to understand the scale of climate change impact on mental health (particularly in vulnerable groups), active public health intervention and system-level changes to support long-term mental health impacts of climate change, and monitoring these impacts through indicators to raise awareness and drive policy change [23].

### 4.7 Limitations

This SOSCHI evidence synthesis has many strengths. The scoping review was rigorously carried out and designed in close collaboration between CPHTG and ONS; it was conducted according to a registered protocol and with the support of an experienced information specialist. Further, data visualizations (Fig. 3, Fig. 4) allow end users (e.g., policy makers and researchers) to better extract insights from this rich data set.

This scoping review also has several limitations. To support study feasibility, our eligibility criteria excluded publications prior to 2017, those only available in a language other than English and those reporting on indirect mental health outcomes from EWEs, still leaving over 3,600 citations. The determination as to whether a mental health outcome was unique was limited by reported details in the included studies and entailed subjective assessments by the review team. To minimize this subjectivity, assessments were verified by a second reviewer during data abstraction. A challenge in the data was the lack of granularity with regards to the reported age, gender and race/ethnicity of included populations. While the included studies reported mental health outcomes associated with EWEs, we cannot definitively attribute the EWEs to climate change. This is a universal challenge in climate change research. However, there is a growing body of evidence supporting the relationship between climate change and EWE frequency, duration and intensity and recent transdisciplinary guidance documents on attribution of human health outcomes to climate change [1,24]. It was outside of our scope to evaluate and report on psychometric properties. Future work is needed on characterizing the psychometric properties across these 130 measures of mental health impact.

### 4.8 Directions for future research

We identified studies originating from 29 countries. LMICs are well represented in our sample, contributing diversity across national social, economic and climatic contexts [25]. This international representation is important to help map the impact of EWEs on mental health across regions, particularly since different parts of the world experience different types, frequency or intensity of EWEs. However, it is important to note that, given the markedly greater climate impacts in these regions, LMICs may not be well-represented based on total burden of EWEs. The problems of statistical capacity and limited data availability in LMICs are central considerations, and in future work it would be beneficial to increase the availability of data on mental health outcomes across different regions and contexts. In turn, this may help to inform preventive population health and clinical strategies. For example, identifying countries or regions with a high occurrence of EWEs and where available data show a low frequency of mental health outcome may help to uncover effective local or national policies, mental health systems and services that have allowed the country to be more climate change resilient when it comes to mental health.

## 5 Conclusions

SOSCHI is leveraging evidence synthesis for data-informed official statistics relevant to climate change and health. Indicators for how climate change impacts mental health must be based on recent and rigorous evidence synthesis, given that the amount of research in mental health and climate change is expanding rapidly. This scoping review contributes a global overview of current reporting practices and our findings support a need for standardization to enable global comparisons and decision-making. The SOSCHI project encompasses further work, building on our findings, via critical evaluation with policy end-users from low-, middle- and high-income countries; it is anticipated that this engagement will improve the generalizability and utility of climate change mental health indicators across different international contexts.

## CRediT authorship contribution statement

**Denise Thomson:** Writing – review & editing, Writing – original draft, Supervision, Project administration, Formal analysis, Conceptualization. **Meghan Sebastianski:** Writing – review & editing, Writing – original draft, Visualization, Supervision, Project administration, Formal analysis, Data curation, Conceptualization. **Katie O’Hearn:** Writing – review & editing, Writing – original draft, Project administration, Formal analysis. **Megan Nuspl:** Writing – original draft, Visualization, Formal analysis, Data curation. **Sol Bobier:** Visualization, Formal analysis, Data curation. **Sami Aftab Abdul:** Visualization, Formal analysis. **Matt Pearce:** Writing – review & editing, Formal analysis, Conceptualization. **Vijendra Ingole:** Writing – review & editing, Formal analysis, Conceptualization. **Myer Glickman:** Writing – review & editing, Funding acquisition, Formal analysis, Conceptualization. **Richard J Webster:** Writing – review & editing, Writing – original draft, Supervision, Project administration, Formal analysis, Conceptualization.

## Declaration of competing interest

The authors declare that they have no known competing financial interests or personal relationships that could have appeared to influence the work reported in this paper.

## Appendix Supplementary materials

Image, application 1

Image, application 2

Image, application 3

Image, application 4

## Footnotes

Supplementary material associated with this article can be found, in the online version, at doi:10.1016/j.joclim.2026.100743.

## References

[1] Otto F. (2020).

[2] Cissé G., McLeman R., Adams H., Aldunce P., Bowen K., Campbell-Lendrum D., Menne B., Semenov S., Toussaint J.-F. (2002). Climate change 2022: impacts, adaptation and vulnerability.

[3] Fernandez A., Black J., Jones M., Wilson L., Salvador-Carulla L., Astell-Burt T. (2015). Flooding and mental health: a systematic mapping review. PLoS One.

[4] Frumkin H. (2022). Hope, health, and the climate crisis. J Clim Chang Health.

[5] Vins H., Bell J., Saha S., Hess J. (2015). The mental health outcomes of drought: a systematic review and causal process diagram. Int J Env Res Public Health.

[6] Aylward B., Cunsolo A., Vriezen R., Harper S.L. (2022). Climate change is impacting mental health in north America: a systematic scoping review of the hazards, exposures, vulnerabilities, risks and responses. Int Rev Psychiatry.

[7] Thomson D., Cumpston M., Delgado-Figueroa N., Ebi K.L., Haddaway N., van der Heijden M. (2022). Protecting human health in a time of climate change: how Cochrane should respond. Cochrane Database Syst Rev.

[8] Office for National Statistics (2026). https://www.ons.gov.uk/aboutus/whatwedo/programmesandprojects/standardsforofficialstatisticsonclimatehealthinteractions.

[9] Arksey H., O'Malley L. (2005). Scoping studies: towards a methodological framework. Int J Soc Res Methodol.

[10] Tricco A.C., Lillie E., Zarin W., O'Brien K.K., Colquhoun H., Levac D. (2018). PRISMA extension for scoping reviews (PRISMA-ScR): checklist and explanation. Ann Intern Med.

[11] Wellcome. Low- and middle-income countries [Internet]. London (UK): Wellcome. [cited 2026 Jun 23]. Available from: https://wellcome.org/research-funding/guidance/prepare-to-apply/low-and-middle-income-countries.

[12] Pearce M., Watkins E., Glickman M., Lewis B., Ingole V. (2024). https://zenodo.org/records/14050225.

[13] Karabegovic A., Indermaur E., Santi S., Fierz K. (2025). Prevalence of psychiatric symptoms and mental health disorders in the home care population: a systematic literature review. J Community Health Nurs.

[14] Borkenhagen D., Messier G., Masrani T., Gunn E., Bahji A., Barry R. (2025). Prevalence of mental health and substance use disorders among adolescents experiencing homelessness: a systematic review. Community Ment Health J.

[15] Caldarelli G., Pizzini B., Cosenza M., Troncone A. (2024). The prevalence of mental health conditions and effectiveness of psychological interventions among university students in Italy: a systematic literature review. Psychiatry Res.

[16] Pfeffermann D., Sverchkov M. (2025). Use of nonprobability samples for official statistics, state of the art. Surv Methodol.

[17] American Psychological Association (2018). https://www.apa.org/ptsd-guideline/assessment.

[18] Horsky J., Drucker E.A., Ramelson H.Z. (2018). Accuracy and completeness of clinical coding using ICD-10 for ambulatory visits. AMIA Annu Symp Proc.

[19] Nelson S., Yin Y., Rivera E., Shao Y., Ma P., Tuttle M. (2024). Are ICD codes reliable for observational studies? Assessing coding consistency for data quality. Digit Health.

[20] Ahad A., Sanchez-Gonzalez M., Junquera P. (2023). Understanding and addressing mental health stigma across cultures for improving psychiatric care: a narrative review. Cureus.

[21] Otero Varela L., Knudsen S., Carpendale S., Eastwood C., Quan H. (2019). Proceedings of the 10th IEEE workshop on visual analytics in healthcare, VAHC 2019.

[22] Charlson F., Ali S., Augustinavicius J., Benmarhnia T., Birch S., Clayton S. (2022). Global priorities for climate change and mental health research. Env Int.

[23] Cordiner R., Gillingham E., Simmons Z., Morrison K., Tran A., Gopfert A. (2025). https://assets.publishing.service.gov.uk/media/69146cc3db01ecfcf96fc825/climate-change-and-mental-health-full-report.pdf.

[24] Ebi K.L., Haines A., Andrade R.F.S., Åström C., Barreto M.L., Bonell A. (2025). The attribution of human health outcomes to climate change: transdisciplinary practical guidance. Clim Change.

[25] Woods W.A., Watson M., Ranaweera S., Tajuria G., Sumathipala A. (2023). Under-representation of low and middle income countries (LMIC) in the research literature: ethical issues arising from a survey of five leading medical journals: have the trends changed?. Glob Public Health.

