# Supplementary material for: Mental health outcomes relating to climate-sensitive extreme weather events: A scoping review of measures of mental health impact
Source: J Clim Chang Health. 2026 Sep-Oct;31:100743. doi: 10.1016/j.joclim.2026.100743 (PMC13573736; doi:10.1016/j.joclim.2026.100743)
Supplement: Supplementary file 1 [file mmc1.pdf]

Supplementary Material 1. Search Strategy

Database: Ovid MEDLINE(R) ALL <1946 to September 26, 2024>

Search Date: 28 September 2024

----

1 (climate or climatic).hw,tw. (186217)

2 exp climate change/ or (climat* adj5 (adapta* or chang* or crisis or destabil* or emergency or instabil* or related or sensitivit* or unstabl* or variab* or warming)).mp. (102917)

3 greenhouse effect/ or greenhouse effect*.tw,kf,kw,ot. (7155)

4 ((atmospheric* or global or planet*) adj5 (heating or warming)).mp. (18887)

5 or/1-4 (203984)

6 exp weather/ or exp extreme weather/ or weather event*.tw,kf,kw,ot. (594925)

7 ((climat* or natural* or weather*) adj2 (disaster* or hazard*)).tw,kf,kw,ot. (8883)

8 ((climate change or extreme climat* or severe climat*) adj2 event*).tw,kf,kw,ot. (1009)

9 ((disrupt* or extrem* or severe) adj3 (storm* or weather)).tw,kf,kw,ot. (4548)

10 extreme cold/ or exp cold temperature/ or ((disrupt* or extrem* or severe) adj3 (cold or freez*)).tw,kf,kw,ot. (83711)

11 (freez* adj2 (condition* or temperature* or weather*)).tw,kf,kw,ot. (4657)

12 cyclonic storms/ or lightning/ or tornadoes/ or (cyclon* or extratropical or gales or hurricane* or landspout* or land spout* or tornado* or tropical storm* or typhoon* or waterspout* or water spout* or wind storm* or windstorm*).tw,kf,kw,ot. (11481)

13 ((extreme* or gale force or high* or severe*) adj2 wind*).tw,kf,kw,ot. (2447)

14 (convective storm* or derecho* or hail or hailstorm* or lightning or monsoon* or supercell? or super cell? or thunder storm* or thunderstorm*).tw,kf,kw,ot. (12514)

15 droughts/ or (drought* or dust storm* or duststorm* or sand storm* or sandstorm*).tw,kf,kw,ot. (39405)

16 ((declin* or decreas* or low or lower*) adj3 (precipitation or rain? or rainfall*)).tw,kf,kw,ot. (4917)

17 extreme heat/ or hot temperature/ or (extreme heat or heat wave* or heatwave or hot weather or severe heat* or severe weather).tw,kf,kw,ot. (132569)

18 ((high* or hot* or warm*) adj3 temperature*).tw,kf,kw,ot. (129016)

19 floods/ or (flood or flooding or floods or mud flow* or mudflow* or mudslide* or mud slide* or sinkhole* or storm surge*).tw,kf,kw,ot. (21652)

20 exp rain/ or ((chang* or extreme* or heavy or high or higher or increas*) adj3 (precipitation or rain? or rainfall* or storm-induc* or storm surge*)).tw,kf,kw,ot. (25040)

21 (sea level* adj3 (increase or increasing or increases or rise or rises or rising)).tw,kf,kw,ot. (2528)

22 avalanches/ or snow/ or (avalanche? or blizzard? or ice storm* or flash freez* or freezing rain* or polar vortex* or snow debris or snowstorm* or snow storm* or winter storm*).tw,kf,kw,ot. (7435)

23 landslide/ or (landslide* or land slide* or mudslide* or mud slide* or rockslide* or rock slide*).tw,kf,kw,ot. (1543)

24 exp natural disasters/ or natural disaster*.tw,kf,kw,ot. (33545)

25 tsunamis/ or tidal waves/ or (megatsunami* or tsunami* or tidal wave*).tw,kf,kw,ot. (3810)

26 wildfires/ or (brush fire* or brushfire* or bush fire* or bushfire* or fire storm* or firestorm* or forest fire* or pasture fire* or peat fire* or peatland fire* or wildfire* or wild* fire*).tw,kf,kw,ot. (6675)

27 or/6-26 (805669)

28 exp mental disorders/ or mental health/ or (mental* adj3 (diagnos* or disease* or disorder* or health* or hygiene or ill*)).tw,kf,kw,ot. (1722369)

29 (behav* adj2 (disorder* or health condition* or illness*)).tw,kf,kw,ot. (23346)

30 exp anxiety disorders/ or anxiety/ or (anxiet* or neuroses or neurosis or neurotic* or panic attack* or panic disorder* or psychoneuros*).tw,kf,kw,ot. (379451)

31 depression/ or exp mood disorders/ or (depressed or depression or depressive or mood disorder*).tw,kf,kw,ot. (664605)

32 (psychiat* or psychol* or psychopath* or psychot*).tw,kf,kw,ot. (796488)

33 exp stress, psychological/ or exp stress disorders, traumatic/ or (PTSD or stress or stresses or stressful or stressor? or trauma* or posttrauma*).tw,kf,kw,ot. (1666635)

34 exp suicide/ or exp self-injurious behavior/ or (suicid* or self-harm* or self-injur*).tw,kf,kw,ot. (133878)

35 or/28-34 (3915255)

36 systematic review.pt. or systematic reviews as topic/ or (systematic* adj3 (overview* or review*)).mp. (407278)

37 meta-analysis.pt. or meta-analysis as topic/ or (meta-anal* or metaanal* or meta-regression* or metaregression*).mp. (356373)

38 (cinahl or cinhal or cochrane or embase or medline or pubmed or "web of science").ab. (400850)

39 (data adj2 extract*).tw,kf,kw,ot. (105085)

40 (evidence synthes* or gap map*).mp. (9073)

41 ((methodologic* or quantitative) adj3 (overview* or review*)).mp. (10666)

42 ((rapid or scoping or umbrella) adj3 (review* or systematic)).mp. (37934)

43 observational study/ or observational studies as topic/ or (observational adj4 (analys* or design* or studies or study)).mp. (325783)

44 case-control studies/ or (case adj2 (compar* or control or controlled or series)).mp. (525529)

45 cohort analysis/ or cohort studies/ or cohort*.mp. (1087374)

46 cross-sectional studies/ or ((cross-sectional or crosssectional) adj5 (analys* or design or studies or study or survey or trial)).tw,kf,kw,ot. (664801)

47 prevalence.ti. or (prevalence adj5 (analys* or studies or study)).tw,kf,kw,ot. (268527)

48 (quasiexperiment* or quasi experiment*).tw,kf,kw,ot. (24527)

49 or/36-48 (3109313)

50 5 and 27 and 35 and 49 (588)

51 exp animals/ not humans/ (5262597)

52 50 not 51 (535)

53 limit 52 to yr="2007 -Current" (520)

Database: Embase Classic+Embase <1947 to 2024 September 27>

Search Date: 28 September 2024

----

1 (climate or climatic).hw,tw. (210044)

2 exp climate change/ or (climat* adj5 (adapta* or chang* or crisis or destabil* or emergency or instabil* or related or sensitivit* or unstabl* or variab* or warming)).mp. (114305)

3 greenhouse effect/ or greenhouse effect*.tw,kf,kw,ot. (20359)

4 ((atmospheric* or global or planet*) adj5 (heating or warming)).mp. (17332)

5 or/1-4 (227194)

6 exp weather/ or exp severe weather/ or weather event*.tw,kf,kw,ot. (79352)

7 ((climat* or natural* or weather*) adj2 (disaster* or hazard*)).tw,kf,kw,ot. (9657)

8 ((climate change or extreme climat* or severe climat*) adj2 event*).tw,kf,kw,ot. (944)

9 ((disrupt* or extrem* or severe) adj3 (storm* or weather)).tw,kf,kw,ot. (4777)

10 ((disrupt* or extrem* or severe) adj3 (cold or freez*)).tw,kf,kw,ot. (4276)

11 (freez* adj2 (condition* or temperature* or weather*)).tw,kf,kw,ot. (5058)

12 hurricane/ or lightning/ or tornado/ or (cyclon* or extratropical or gales or hurricane* or landspout* or land spout* or tornado* or tropical storm* or typhoon* or waterspout* or water spout* or wind storm* or windstorm*).tw,kf,kw,ot. (15503)

13 ((extreme* or gale force or high* or severe*) adj2 wind*).tw,kf,kw,ot. (2887)

14 (convective storm* or derecho* or hail or hailstorm* or lightning or monsoon* or supercell? or super cell? or thunder storm* or thunderstorm*).tw,kf,kw,ot. (14314)

15 drought/ or (drought* or dust storm* or duststorm* or sand storm* or sandstorm*).tw,kf,kw,ot. (37146)

16 ((declin* or decreas* or low or lower*) adj3 (precipitation or rain? or rainfall*)).tw,kf,kw,ot. (5105)

17 (extreme heat or heat wave* or heatwave or hot weather or severe heat* or severe weather).tw,kf,kw,ot. (7526)

18 ((high* or hot* or warm*) adj3 temperature*).tw,kf,kw,ot. (123722)

19 flooding/ or (flood or flooding or floods or mud flow* or mudflow* or mudslide* or mud slide* or sinkhole* or storm surge*).tw,kf,kw,ot. (25515)

20 rain/ or ((chang* or extreme* or heavy or high or higher or increas*) adj3 (precipitation or rain? or rainfall* or storm-induc* or storm surge*)).tw,kf,kw,ot. (37718)

21 (sea level* adj3 (increase or increasing or increases or rise or rises or rising)).tw,kf,kw,ot. (2386)

22 avalanche/ or snow/ or (avalanche? or blizzard? or ice storm* or flash freez* or freezing rain* or polar vortex* or snow debris or snowstorm* or snow storm* or winter storm*).tw,kf,kw,ot. (9301)

23 landslide/ or (landslide* or land slide* or mudslide* or mud slide* or rockslide* or rock slide*).tw,kf,kw,ot. (1648)

24 natural disaster/ or natural disaster*.tw,kf,kw,ot. (9610)

25 tsunami/ or (megatsunami* or tsunami* or tidal wave*).tw,kf,kw,ot. (4741)

26 wildfire/ or (brush fire* or brushfire* or bush fire* or bushfire* or fire storm* or firestorm* or forest fire* or pasture fire* or peat fire* or peatland fire* or wildfire* or wild* fire*).tw,kf,kw,ot. (7599)

27 or/6-26 (315093)

28 exp mental disease/ or exp mental health/ or (mental* adj3 (diagnos* or disease* or disorder* or health* or hygiene or ill*)).tw,kf,kw,ot. (3252453)

29 (behav* adj2 (disorder* or health condition* or illness*)).tw,kf,kw,ot. (34112)

30 exp anxiety disorder/ or anxiety/ or (anxiet* or neuroses or neurosis or neurotic* or panic attack* or panic disorder* or psychoneuros*).tw,kf,kw,ot. (740319)

31 depression/ or exp mood disorder/ or (depressed or depression or depressive or mood disorder*).tw,kf,kw,ot. (1090538)

32 (psychiat* or psychol* or psychopath* or psychot*).tw,kf,kw,ot. (1111169)

33 exp mental stress/ or exp posttraumatic stress disorder/ or (PTSD or stress or stresses or stressful or stressor? or trauma* or posttrauma*).tw,kf,kw,ot. (2199748)

34 exp suicide/ or (suicid* or self-harm* or self-injur*).tw,kf,kw,ot. (165562)

35 or/28-34 (5735561)

36 systematic review/ or (systematic* adj3 (overview* or review*)).mp. (645475)

37 exp meta analysis/ or (meta-anal* or metaanal* or meta-regression* or metaregression*).mp. (511377)

38 (cinahl or cinhal or cochrane or embase or medline or pubmed or "web of science").ab. (499565)

39 (data adj2 extract*).tw,kf,kw,ot. (142035)

40 (evidence synthes* or gap map*).mp. (10064)

41 ((methodologic* or quantitative) adj3 (overview* or review*)).mp. (12278)

42 ((rapid or scoping or umbrella) adj3 (review* or systematic)).mp. (40425)

43 observational study/ or observational studies as topic/ or (observational adj4 (analys* or design* or studies or study)).mp. (544899)

44 exp case control study/ or (case adj2 (compar* or control or controlled or series)).mp. (497758)

45 cohort analysis/ or cohort*.mp. (1918387)

46 cross-sectional study/ or ((cross-sectional or crosssectional) adj5 (analys* or design or studies or study or survey or trial)).mp. (829945)

47 prevalence.ti. or (prevalence adj5 (analys* or studies or study)).mp. (402340)

48 quasi experimental study/ or (quasiexperiment* or quasi experiment*).mp. (32507)

49 or/36-48 (4491426)

50 5 and 27 and 35 and 49 (590)

51 (exp animal/ or animal.hw. or nonhuman/) not (exp human/ or human cell/ or (human or humans).ti.) (8253987)

52 50 not 51 (521)

53 limit 52 to yr="2007 -Current" (515)

Database: APA PsycInfo <1806 to September 2024 Week 4>

Search Date: 28 September 2024

----

1 (climate or climatic).hw,tw. (46781)

2 exp climate change/ or (climat* adj5 (adapta* or chang* or crisis or destabil* or emergency or instabil* or related or sensitivit* or unstabl* or variab* or warming)).mp. (10243)

3 greenhouse effect*.mp. (117)

4 global warming/ or ((atmospheric* or global or planet*) adj5 (heating or warming)).mp. (1050)

5 or/1-4 (47414)

6 atmospheric conditions/ or exp extreme weather/ or weather.mp. (5758)

7 ((climat* or natural*) adj2 (disaster* or hazard*)).mp. (8608)

8 ((climate change or extreme climat* or severe climat*) adj2 event*).mp. (55)

9 ((disrupt* or extrem* or severe) adj3 (cold or freez*)).mp. (239)

10 (freez* adj2 (condition* or event* or temperature*)).mp. (600)

11 (cyclon* or extratropical or gales or hurricane* or landspout* or land spout* or tornado* or tropical storm* or typhoon* or waterspout* or water spout* or wind storm* or windstorm*).mp. (3558)

12 ((extreme* or gale force or high* or severe*) adj2 wind*).mp. (210)

13 (convective or derecho* or hail or hailstorm* or lightning or monsoon* or supercell? or super cell? or thunderstorm*).mp. (1264)

14 (drought* or duststorm* or sandstorm*).mp. (600)

15 ((declin* or decreas* or low or lower*) adj3 (precipitation or rain? or rainfall*)).mp. (81)

16 (extreme heat or heat wave* or heatwave or severe heat*).mp. (293)

17 ((high* or hot* or warm*) adj3 temperature*).mp. (4869)

18 (flood or flooding or floods or mud flow* or mudflow* or mudslide* or mud slide* or sinkhole*).mp. (3032)

19 ((chang* or extreme* or heavy or high or higher or increas*) adj3 (precipitation or rain? or rainfall*)).mp. (275)

20 (sea level* adj3 (increase or increasing or increases or rise or rises or rising)).mp. (100)

21 (avalanche? or blizzard? or flash freez* or freezing rain* or polar vortex* or snow debris or snowstorm*).mp. (411)

22 (landslide* or land slide* or mudslide* or mud slide* or rockslide* or rock slide*).mp. (155)

23 (megatsunami* or tsunami* or tidal wave*).mp. (1188)

24 natural disasters/ or natural disaster*.mp. (8345)

25 (megatsunami* or tsunami* or tidal wave*).mp. (1188)

26 storm*.mp. (3828)

27 (wildfire* or wild* fire* or brush fire* or brushfire* or bush fire* or bushfire* or firestorm* or forest fire* or pasture fire* or peat fire* or peatland fire*).mp. (875)

28 or/6-27 (28893)

29 exp mental disorders/ or exp mental health/ or (mental* adj3 (diagnos* or disease* or disorder* or health* or hygiene or ill*)).mp. (1386388)

30 (behav* adj2 (disorder* or health condition* or illness*)).mp. (46956)

31 exp anxiety disorders/ or exp anxiety/ or (anxiet* or neuroses or neurosis or neurotic* or panic attack* or panic disorder* or psychoneuros*).mp. (361242)

32 depression.hw. or exp mood disorders/ or (depressed or depression or depressive or mood disorder*).mp. (465577)

33 (psychiat* or psychol* or psychopath* or psychot*).mp. (1547945)

34 exp psychological stress/ or exp stress/ or (PTSD or stress or stresses or stressful or stressor? or trauma* or posttrauma*).mp. (501126)

35 exp suicidal behavior/ or exp nonsuicidal self-injury/ or (suicid* or self-harm* or self-injur*).mp. (97445)

36 temperature effects/ or (temperature adj3 effect?).mp. (3989)

37 or/29-36 (2649804)

38 "systematic review"/ or (systematic* adj3 (overview* or review*)).mp. (63813)

39 meta analysis/ or (meta-anal* or metaanal* or meta-regression* or metaregression*).mp. (59244)

40 (cinahl or cinhal or cochrane or embase or medline or pubmed or "web of science").ab. (43914)

41 (data adj2 extract*).mp. (10922)

42 (evidence synthes* or gap map*).mp. (1257)

43 ((methodologic* or quantitative) adj3 (overview* or review*)).mp. (4987)

44 ((rapid or scoping or umbrella) adj3 (review* or systematic)).mp. (8646)

45 (observational adj4 (analys* or design* or studies or study)).mp. (23062)

46 (case adj2 (compar* or control or controlled or series)).mp. (47747)

47 cohort analysis/ or cohort studies/ or cohort*.mp. (119937)

48 ((cross-sectional or crosssectional) adj5 (analys* or design or studies or study or survey or trial)).mp. (134649)

49 prevalence.ti. or (prevalence adj5 (analys* or studies or study)).mp. (40966)

50 quasi experimental methods/ or (quasiexperiment* or quasi experiment*).mp. (18485)

51 or/38-50 (458790)

52 5 and 28 and 37 and 51 (104)

53 limit 52 to yr="2007 -Current" (91)

Database: Clarivate Web of Science (SCI-Expanded, CPCI-S, ESCI)

Search Date: 28 September 2024

#8 #7 Timespan: 2007-01-01 to 2024-10-28 1006

#7 #1 AND (#2 OR #3) AND #4 AND (#5 OR #6) 1025

#6 TS=(((cross-sectional OR crosssectional OR observational) NEAR/5 (analys* OR design OR studies OR study OR survey OR trial))) OR TI=(prevalence) OR TS=((prevalence NEAR/5 (analys* OR studies OR study))) 1042557

#5 TS=((systematic* NEAR/3 (overview* OR review*))) OR AB=((cinahl OR cinhal OR Cochrane OR Embase OR medline OR pubmed OR "web of science")) OR TS=(data NEAR/2 extract*) OR TS=((cohort* OR "evidence synthes*" OR "gap map*" OR meta-anal* OR metaanal* OR meta-regression* OR metaregression* OR quasiexperiment*OR "quasi experiment*")) OR TS=(((methodologic* OR quantitative OR rapid OR scoping OR umbrella) NEAR/3 (overview* OR review* OR systematic))) OR TS=((case NEAR/2 (compar* OR control OR controlled OR series))) 2375342

#4 TS=((mental*NEAR/3 (diagnos* OR disease* OR disorder* OR health* OR hygiene OR ill*))) OR TS=((behav* NEAR/2 (disorder* OR "health condition*" OR illness*))) OR TS=((anxiet* OR depressed OR depression OR depressive OR "mood disorder*" OR neuroses OR neurosis OR neurotic* OR "panic attack*" OR "panic disorder*" OR posttrauma* OR psychiat* OR psychol* OR psychoneuros* OR psychopath* OR psychot* OR PTSD OR self-harm* OR self-injur* OR stress OR stresses OR stressful OR stressor* OR suicid* OR trauma*)) 4495472

#3 TS=(((climat* OR natural*) NEAR/2 (disaster*OR hazard*))) OR TS=((("climate change" OR "extreme climat*" OR "severe climat*") NEAR/2 (event*))) OR TS=(((disrupt* OR extrem* OR severe) NEAR/3 (cold OR freez* OR heat OR hot))) OR TS=(((freez* OR high* OR hot* OR warm*) NEAR/3 (condition* OR temperature* OR weather*))) OR TS=(((convective OR extreme* OR "gale force" OR high* OR severe*) NEAR/2 (storm* OR

wind*))) OR TS=(((chang* OR declin* OR decreas* OR extreme* OR heavy OR high OR higher OR increas* or low OR lower*) NEAR/3 (precipitation OR rain* OR rainfall* OR storm-induc* OR "storm surge*"))) OR TS=((("sea level*") NEAR/3 (increase OR increasing OR increases OR rise OR rises OR rising))) 1327735

#2 TS=((weather OR avalanche* OR blizzard* OR "brush fire*" OR brushfire* OR "bush fire*" OR bushfire* OR convective OR cyclon* OR derecho* OR drought* OR "dust storm*" OR duststorm* OR extratropical OR "extreme heat" OR "fire storm*" OR firestorm* OR "flash freez*" OR flood OR flooding OR floods OR "forest fire*" OR "freezing rain*" OR gales OR hail OR hailstorm* OR "heat wave*" OR heatwave OR "hot weather" OR hurricane* OR "ice storm*" OR landspout* OR landslide* OR "land slide*" OR "land spout*" OR lightning OR megatsunami* OR monsoon* OR "mud flow*" OR mudflow* OR mudslide* OR "mud slide*" OR "natural disaster*" OR "pasture fire*" OR "peat fire*" OR "peatland fire*" OR "polar vortex*" OR rockslide* OR "rock slide*" OR "sand storm*" OR sandstorm* OR "severe heat*" OR "severe weather" OR sinkhole* OR "snow debris" OR snowstorm* OR "snow storm*" OR "storm surge*" OR supercell* OR "super cell*" OR "thunder storm*" OR thunderstorm* OR "tidal wave*" OR tornado* OR "tropical storm*" OR tsunami* OR typhoon* OR waterspout* OR "water spout*" OR wildfire* OR "wild* fire*" OR "wind storm*" OR windstorm* OR "winter storm*")) 1000966

#1 TI=(climate) OR TS=((climat* NEAR/5 (adapta* OR chang* OR crisis OR destabil* OR emergency OR instabil* OR related OR sensitivit* OR unstabl* OR variab* OR warming))) OR TS=("greenhouse effect*") OR TS=(((atmospheric* OR global OR planet*) NEAR/5 (heating OR warming))) 619803

Database: Elsevier Scopus

Search Date: 28 September 2024

----

1 INDEXTERMS(climate OR climatic OR "climate change" OR "global warming" OR "greenhouse effect") OR TITLE-ABS-KEY(climat* W/5 (adapta* OR chang* OR crisis OR destabil* OR emergency OR instabil* OR related OR sensitivit* OR unstabl* OR variab* OR warming)) OR TITLE-ABS-KEY("greenhouse effect") OR TITLE-ABS-KEY((atmospheric* OR global OR planet*) W/5 (heating OR warming)) 889,755

2 INDEXTERMS("extreme weather" OR weather OR avalanches OR "cold temperature*" OR "cyclonic storms" OR drought* OR "extreme cold" OR "extreme heat" OR floods OR "hot temperature*" OR landslide* OR lightning OR "natural disasters" OR rain OR sandstorm* OR snow OR tornadoes OR "tidal waves" OR tsunamis OR wildfire*) OR TITLE-ABS-KEY(weather OR avalanche* OR blizzard* OR "brush fire*" OR brushfire* OR "bush fire*" OR bushfire* OR convective OR cyclon* OR derecho* OR extratropical OR "extreme heat" OR "fire storm*" OR firestorm* OR "flash freez*" OR flood* OR "forest fire*" OR "freezing rain*" OR gale* OR hail OR hailstorm* OR "heat wave*" OR heatwave OR hurricane* OR landslide* OR "land slide*" OR landspout* OR "land spout*" OR lightning OR megatsunami* OR monsoon* OR "mud flow*" OR mudflow* OR mudslide* OR "mud slide*" OR "natural disaster*" OR "pasture fire*" OR "peat fire*" OR "peatland fire*" OR "polar vortex*" OR rockslide* OR "rock slide*" OR "severe heat*" OR sinkhole* OR "snow debris" OR snowstorm* OR storm* OR supercell* OR "super cell*" OR thunderstorm* OR "tidal wave*" OR tornado* OR tsunami* OR typhoon* OR waterspout* OR "water spout*" OR wildfire* OR "wild fire*" OR windstorm*) OR TITLE-ABS-KEY((climat* OR natural*) W/2 (disaster* OR hazard*)) OR TITLE-ABS-KEY(("climate change" OR "extreme climat*" OR "severe climat*") W/2 event*) OR TITLE-ABS-KEY((disrupt* OR extrem* OR severe) W/3 (cold OR freez* OR heat* OR hot* OR warm*)) OR TITLE-ABS-KEY((declin* OR decreas* OR low OR lower*) W/3 (precipitation OR rain* OR rainfall*)) OR TITLE-ABS-KEY((freez* OR high* OR hot* OR warm*) W/3 (condition* OR temperature*)) OR TITLE-ABS-KEY((extreme* OR high* OR severe*) W/2 wind*) OR TITLE-ABS-KEY((chang* OR extreme*

OR heavy OR high OR higher OR increas*) W/3 (precipitation OR rain* OR rainfall*)) OR TITLE-ABS-KEY("sea level*" W/3 (increas* OR rise* OR rising)) 3,171,719

3 INDEXTERMS("mental disorders" OR "mental health" OR "mood disorders" OR depression OR anxiety OR "anxiety disorders" OR "psychological stress" OR stress OR suicide OR "self-injurious behavior" OR "stress disorder") OR TITLE-ABS-KEY(mental* W/3 (diagnos* OR disease* OR disorder* OR health* OR hygiene OR ill*)) OR TITLE-ABS-KEY(behav* W/2 (disorder* OR "health condition*" OR illness*)) OR TITLE-ABS-KEY(anxiet* OR depressed OR depression OR depressive OR "mood disorder*" OR neuroses OR neurosis OR neurotic* OR "panic attack*" OR "panic disorder*" OR posttrauma* OR psychiat* OR psychol* OR psychopath* OR psychot* OR psychoneuros* OR PTSD OR stress OR stresses OR stressful OR stressor* OR self-harm* OR self-injur* OR suicid* OR trauma*) 7,618,029

4 DOCTYPE(meta-analysis OR "systematic review") OR INDEXTERMS("case-control studies" OR "cohort analysis" OR "cohort studies" OR "cross-sectional studies" OR "meta-analysis as topic" OR "observational study" OR "observational studies as topic" OR "systematic reviews as topic") OR TITLE-ABS-KEY(cohort* OR "evidence synthes*" OR "gap map*" OR meta-anal* OR metaanal* OR meta-regression* OR metaregression* OR observational OR quasiexperiment* OR "quasi experiment*") OR TITLE-ABS-KEY(systematic* W/3 (overview* OR review*)) OR TITLE-ABS-KEY((rapid OR scoping OR umbrella) W/3 (review* OR systematic)) OR TITLE-ABS-KEY(data W/2 extract*) OR TITLE-ABS-KEY((methodologic* OR quantitative) W/3 (overview* OR review*)) OR TITLE-ABS-KEY(case W/2 (compar* OR control OR controlled OR series)) OR TITLE-ABS-KEY((cross-sectional OR crosssectional) W/5 (analys* OR design OR studies OR study OR survey OR trial)) OR TITLE-ABS-KEY(observational W/4 (analys* OR design* OR studies OR study)) OR TITLE-ABS-KEY(prevalence W/5 (analys* OR studies OR study)) OR TITLE(prevalence) OR ABS(cinahl OR cinhal OR cochrane OR embase OR medline OR pubmed OR "web of science") 4,349,023

5 1 AND 2 AND 3 AND 4 1,366

6 LIMIT 5 TO PUBYEAR > 2006 AND PUBYEAR < 2025 1,320

Database: EBSCO CABI Global Health

Search Date: 28 September 2024

S28 S27 Limiters - Publication Year: 20070101-20241031 731

S27 S5 AND S14 AND S26 751

S26 S19 OR S20 OR S21 OR S22 OR S23 OR S24 OR S25 629,866

S25 TX ((cross-sectional OR crosssectional OR observational) N5 (analys* OR design OR studies OR study OR survey OR trial)) 239,492

S24 (TI prevalence N5 (analys* OR studies OR study))) OR (AB prevalence N5 (analys* OR studies OR study))) 75,135

S23 TX (systematic* N3 (overview* OR review*)) 82,459

S22 TX (data N2 extract*) 21,414

S21 TX (cohort* OR "evidence synthes*" OR "gap map*" OR meta-anal* OR metaanal* OR meta-regression* OR metaregression* OR quasiexperiment*OR "quasi experiment*") 257,735

S20 TX (case N2 (compar* OR control OR controlled OR series)) 106,297

S19 TX ((methodologic* OR quantitative OR rapid OR scoping OR umbrella) N3 (overview* OR review* OR systematic)) 10,634

S18 S15 OR S16 OR S17 649,268

S17 TX (anxiet* OR depressed OR depression OR depressive OR "mood disorder*" OR neuroses OR neurosis OR neurotic* OR "panic attack*" OR "panic disorder*" OR posttrauma* OR psychiat* OR psychol* OR psychoneuros* OR psychopath* OR psychot* OR PTSD OR self-harm* OR self-injur* OR stress OR stresses OR stressful OR stressor* OR suicid* OR trauma*) 622,705

S16 TX (behav* N2 (disorder* OR "health condition*" OR illness*)) 12,305

S15 TX (mental* N3 (diagnos* OR disease* OR disorder* OR health* OR hygiene OR ill*)) 109,983

S14 S6 OR S7 OR S8 OR S9 OR S10 OR S11 OR S12 OR S13 53,801

S13 TX ("sea level*") N3 (increase OR increasing OR increases OR rise OR rises OR rising)) 387

S12 TX ((chang* OR declin* OR decreas* OR extreme* OR heavy OR high OR higher OR increas* or low OR lower*) N3 (precipitation OR rain* OR rainfall* OR storm-induc* OR "storm surge*")) 6,551

S11 TX ((convective OR extreme* OR "gale force" OR high* OR severe*) N2 (storm* OR wind*)) 934

S10 TX ((freez* OR high* OR hot* OR warm*) N3 (condition* OR temperature* OR weather*)) 37,095

S9 TX ((disrupt* OR extrem* OR severe) N3 (cold OR freez* OR heat OR hot)) 2,017

S8 TX (("climate change" OR "extreme climat*" OR "severe climat*") N2 (event*)) 322

S7 TX ((climat* OR natural*) N2 (disaster*OR hazard*)) 22

S6 TX (("extreme weather" OR avalanche* OR blizzard* OR "brush fire*" OR brushfire* OR "bush fire*" OR bushfire* OR convective OR cyclon* OR derecho* OR drought* OR "dust storm*" OR duststorm* OR extratropical OR "extreme heat" OR "extreme temperature*" OR "fire storm*" OR firestorm* OR freez*" OR flood*s OR "forest fire*" OR "freezing rain*" OR gales OR hail OR hailstorm* OR "heat wave*" OR heatwave OR "hot weather" OR hurricane* OR "ice storm*" OR landspout* OR landslide* OR "land slide*" OR "land spout*" OR lightning OR megatsunami* OR monsoon* OR "mud flow*" OR mudflow* OR mudslide* OR "mud slide*" OR "natural disaster*" OR "pasture fire*" OR "peat fire*" OR "peatland fire*" OR "polar vortex*" OR rockslide* OR "rock slide*" OR "sand storm*" OR sandstorm* OR "severe heat*" OR "severe weather" OR sinkhole* OR "snow debris" OR snowstorm* OR "snow storm*" OR "storm surge*" OR supercell* OR "super cell*" OR "thunder storm*" OR thunderstorm* OR "tidal wave*" OR tornado* OR "tropical storm*" OR tsunami* OR typhoon* OR waterspout* OR "water spout*" OR wildfire* OR "wild* fire*" OR "wind storm*" OR windstorm* OR "winter storm*")) 10,835

S5 S1 OR S2 OR S3 OR S4 47,664

S4 TX (((atmospheric* OR global OR planet*) N5 (heating OR warming))) 3,815

S3 TX greenhouse effect* 572

S2 TX ((climat* N5 (adapta* OR chang* OR crisis OR destabil* OR emergency OR instabil* OR related OR sensitivit* OR unstabl* OR variab* OR warming))) 26,405

S1 TI ((climate OR climatic)) OR AB ((climate OR climatic)) 42,569

Database: Epistemonikos

Search Date: 29 September 2024

#1 (title:((climate* OR climatic OR greenhouse OR "global warming")) OR abstract:((climate* OR climatic OR greenhouse OR "global warming"))) AND (title:((avalanche* OR blizzard* OR disaster* OR "extreme cold" OR "extremely cold" OR "extreme heat" OR "extremely hot" OR "extreme temperatures" OR fire* OR freezing OR "heat wave" OR "heat waves" OR "heavy rain" OR "heavy rains" OR hurricane* OR ice OR rainfall OR storm* OR snow* OR tornado* OR torrential OR weather OR wildfire*)) OR abstract:((avalanche* OR blizzard* OR disaster* OR "extreme cold" OR "extremely cold" OR "extreme heat" OR "extremely hot" OR "extreme temperatures" OR fire* OR freezing OR "heat wave" OR "heat waves" OR "heavy rain" OR "heavy rains" OR hurricane* OR ice OR rainfall OR storm* OR snow* OR tornado* OR torrential OR weather OR wildfire*))) AND (title:((mental* OR anxiety OR depress* OR mood* OR self-harm* OR self-injur* OR stress* OR suicid*)) OR abstract:((mental* OR anxiety OR depress* OR mood* OR self-harm* OR self-injur* OR stress* OR suicid*))) 317

#2 Limit 1 to publication year 2007-2025 312
