# Supplementary material for: Mental health outcomes relating to climate-sensitive extreme weather events: A scoping review of measures of mental health impact
Source: J Clim Chang Health. 2026 Sep-Oct;31:100743. doi: 10.1016/j.joclim.2026.100743 (PMC13573736; doi:10.1016/j.joclim.2026.100743)
Supplement: Supplementary file 2 [file mmc2.pdf]

Supplementary Material 2 - Table S1. Characteristics of included studies

First Author, Year	Country of study Urban/Rural	Study design	Population (N)	Type of extreme weather event	Mental health outcome(s) evaluated	Measure(s) of mental health impact used
Adams 2021	Ghana<br>Urban	Qualitative photovoice study	Adult residents of Old Fadama<br>N=20	Flooding	Acute anxiety, chronic stress	Self-reported
Agyapong 2018	Canada<br>Urban	Cross-sectional	Adults<br>N=486	Wildfire	Anxiety, nicotine dependence, substance abuse (alcohol and drugs)	Questionnaires
Agyapong 2019/2021	Canada<br>Urban	Cross-sectional	Adults<br>N=486	Wildfire	PTSD	Questionnaire
Agyapong 2020	Canada<br>Urban	Cross-sectional	Adults<br>N=197	Wildfire	GAD, MDD, PTSD, substance abuse	Multiple patient assessment tools (checklist, tests, questionnaire)
Alam 2022	Bangladesh<br>NR	Retrospective	Deaths<br>N=NR	Drought	Suicide deaths (intentional self-harm)	Vital statistics database
An 2018a	China<br>Urban	Cross-sectional	Adolescents exposed to tornado<br>N=443	Tornado	Academic burnout, dispositional mindfulness, parental attachment, PTSD	Multiple patient assessment tools (inventory, scales, questionnaire)
An 2018b	China<br>Urban	Longitudinal	Adolescents<br>N=247	Tornado	Mindfulness, PTG, PTSS	Multiple patient assessment tools (inventory, scales)
An 2019a	China<br>Urban	Longitudinal	Adolescents<br>N=154	Tornado	Depression, PTSD	Multiple patient assessment tools (scales)
An 2019b	USA<br>NR	Survey	Adults<br>N=70267	Hurricane	Poor mental health days	Survey
Ashok 2019	India<br>Rural	Cross-sectional	Adults<br>N=302	Flood	PTSD	Interview and patient assessment tool (scale)
Asim 2022	India<br>Both	Cross-sectional	Adults directly exposed to flood<br>N=276	Flood	Depression, GAD, PTSD	Multiple patient assessment tools (checklist, questionnaire)
Austin 2018/2019	Australia<br>Rural	Cohort	Adult farmers<br>N=664	Drought	Community drought-related stress, general psychological distress, personal drought-related stress	Mulitple patient assessment tools (self-report, scale)
Bailie 2022	Australia<br>Rural	Cross-sectional	Residents >16 years<br>N=225	Flood	PTSD, still distressed	Survey

First Author, Year	Country of study Urban/Rural	Study design	Population (N)	Type of extreme weather event	Mental health outcome(s) evaluated	Measure(s) of mental health impact used
Bandla 2019	India<br>Urban	Cross-sectional	Adults exposed to floods<br>N=223	Flood	Anxiety, depression, poor well-being, PTSD, substance abuse	Interview and multiple patient assessment tools (checklist, inventory, scale, self-report)
Begum 2022	USA<br>NR	Database review	Emergency department cases after hurricane who were >65 years old.	Hurricane	ED visit and hospitalizations that relate to anxiety, adjustment disorder, alcohol and substance abuse, mental health, mood disorder, psychosis, and suicide and intentional harm	Administrative data
Belleville 2019	Canada<br>Urban	Cross-sectional	Adult evacuees<br>N=379	Wildfire	Agoraphobia, alcohol use disorder, drug use disorder, GAD, insomnia disorder, MDD, obsessive-compulsive disorder, panic disorder, PTSD, social phobia	Clinical interview and multiple patient assessment tools (index, scale, questionnaire)
Berman 2021	USA<br>Rural	Cohort	Farmers<br>N=498	Drought	Occupational psychosocial stress ("job strain")	Survey
Bevilacqua 2020	USA<br>Urban	Pilot cross-sectional	Houston residents<br>N=161	Hurricane	Alcohol misuse, anxiety, depression, perceived stress, PTSD, recreational drug use	Multiple patient assessment tools (checklist, scale, questionnaire, self-report)
Binet 2021	Canada<br>Urban	Cross-sectional	Adult evacuees<br>N=1510	Wildfire	Depression, insomnia, mental health service utilisation, PTSD	Multiple patient assessment tools (checklist, index, questionnaires)
Bistricky 2019	USA<br>NR	Cross-sectional	Adult survivors<br>N=801	Hurricane	Depression, PTSS	Multiple patient assessment tools (scale, questionnaire)
Block 2019	Australia<br>Rural	Mixed-methods	Adults<br>N=1056	Wildfire	Depression, psychological distress, PTSD	Multiple patient assessment tools (scale, checklist, questionnaire)
Bozick 2021	USA<br>Urban	Cohort	Adults before and after Hurricane Harvey<br>N=5694	Hurricane	Days a month of poor physical or mental health	Survey
Bromet 2017	USA<br>Urban	Cohort	Responders to Hurricane Sandy<br>N=870	Hurricane	MDD, PTSD	Multiple patient assessment tools (checklist, questionnaire)
Brown 2019	Canada<br>Urban	Comparative cohort	Adolescents<br>N=5866	Wildfire	Alcohol misuse, anxiety, depression, PTSD, substance misuse, suicidality, tobacco use	Multiple patient assessment tools (scale, questionnaire)
Bryant 2017a	Australia<br>NR	Cohort	Adults<br>N=500	Wildfire	Avoidant attachment style, PTSD	Multiple patient assessment tools (scale, checklist)

First Author, Year	Country of study Urban/Rural	Study design	Population (N)	Type of extreme weather event	Mental health outcome(s) evaluated	Measure(s) of mental health impact used
Bryant 2017b	Australia<br>NR	Cohort	Adults<br>N=558	Wildfire	Depression, PTSD	Multiple patient assessment tools (checklist, questionnaire)
Bryant 2018	Australia<br>NR	Cohort	Adults >18 years old<br>N=1017	Wildfire	Fire-related PTSD, major depressive episodes, problem alcohol use, psychological distress, PTSD	Multiple patient assessment tools (checklist, scale, questionnaire)
Bryant 2020	Australia<br>NR	Cohort	Adults<br>N=525	Wildfire	Major depressive episodes, problem alcohol use, psychological distress, PTSD	Multiple patient assessment tools (checklist, questionnaire, scale, test)
Bundo 2021	Switzerland<br>Urban	Time-series	Hospitalizations due to mental disorders<br>N=88996	Extreme heat	Hospitalizations relating to adult and behavioural personality disorder, developmental disorder, mental health, mental retardation, mood disorder, neurotic disorder, organic mental disorder, psychoactive, schizophrenia	Administrative data
Carleton 2017	India<br>Rural	Administrative	Suicide records from India's National Crime Records Bureau<br>N= NR	Extreme heat	Suicides per 100,000	Administrative data
Carlsen 2019	Sweden<br>Urban	Case-crossover	Psychiatric emergency visits<br>N=84230	Extreme temperatures	Psychiatric emergency visits	Administrative data
Casas 2021/2022	Belgium<br>Urban	Case-crossover	Suicide deaths aged >5 years<br>N= 1891	Extreme heat	Suicide mortality	Administrative data
Chau 2020	China<br>Urban	Retrospective	Adults >65 years<br>N=7944	Hot weather	Suicides (violent and non-violent)	Administrative data
Cherbuin 2024	Australia<br>Urban	Cross-sectional	Pregnant and postpartum women<br>N=919	Bushfire	Anxiety, depression, stress	Survey
Cherry 2017	Canada<br>Urban	Cross-sectional	Workers in Fort McMurray<br>N=130	Wildfire	Anxiety, depression	Survey
Chung 2017	Pakistan<br>NR	Cross-sectional	Flood victims<br>N=154	Flood	Dysfunctional cognitions, general psychiatric morbidity, PTSD	Interview and patient assessment tool (scales, questionnaire)

First Author, Year	Country of study Urban/Rural	Study design	Population (N)	Type of extreme weather event	Mental health outcome(s) evaluated	Measure(s) of mental health impact used
Cloud 2023	USA<br>NR	Longitudinal	Incarcerated men<br>N=NR	Extreme heat	Suicide watch incidents	Administrative data
Connon 2021	UK<br>Both	Qualitative ethnographic research	People with and without disabilities<br>N=89	Winter storms	Emotional upheaval	Qualitative interview
Cooper 2019	Ethiopia<br>Rural	Qualitative	Adults from three villages<br>N=64	Drought/water stress	Anxiety (negative-active), depression (negative, active)	Focus groups and interviews
Cortes 2023	USA (Puerto Rico)<br>NR	Cohort	Adults >18 with access to landlines or cellular telephones<br>N= 24555	Hurricane	Binge drinking in the past year, depression	Self-report
Cowlishaw 2021	Australia<br>Rural	Cross-sectional	Adults affected by bushfire<br>N=736	Wildfire	Aggressive behaviours, anger, depression, PTSD, severe mental illness, suicidal ideation, violence exposure, well-being	Multiple patient assessment tools (checklist, scales, self-report, questionnaire)
Crombach 2018	Burundi<br>Urban	Feasibility trial	Adults<br>N= 51	Flood	Depression, PTSD, suicidal tendency	Diagnostic interview and patient assessment tool (questionnaire)
Culqui 2017	Spain<br>Urban	Longitudinal	Emergency admissions due to Alzheimer's disease<br>N=1183	Heatwaves	Alzheimer's disease urgent hospital admissions	Administrative data
Dai 2017a	China<br>Rural	Cross-sectional	Children exposed to flood<br>N=325	Flood	Anxiety, PTSD	Interview and multiple patient assessment tools (checklist, scale)
Dang 2022	Vietnam<br>Urban	Admission data modelling	Hospitalizations<br>N= 7780	Extreme heat	Admissions relating to complex behavioural disorders, disorders of adult personality and behaviour, mental and behavioural disorders, mental retardation, mood (affective) disorders, Neurotic, stress-related and somatoform disorders, schizophrenia, schizotypal and delusional disorders	Administrative admissions data
Dodd 2018	Canada<br>Rural	Qualitative-Phenomenological interview	Adults<br>N=30	Wildfire	Mental and emotional well-being, physical activity and well-being, separation from the land and traditional activities, stories of adaptation and resilience	Interview
Ducy 2021	USA<br>NR	Qualitative study	Parents of children and youth with disabilities impacted by wildfire<br>N= 14	Wildfire	Grief, stress	Interview

First Author, Year	Country of study Urban/Rural	Study design	Population (N)	Type of extreme weather event	Mental health outcome(s) evaluated	Measure(s) of mental health impact used
Edwards 2024	Australia<br>NR	Longitudinal	Adolescents<br>N=4983	Fire, floods, drought	Self-harm, suicidal ideation, suicide attempts	Multiple patient assessment tools (survey, questionnaire)
Eisele 2021	Germany<br>Urban	Admission data	Psychiatric hospital admissions from 2007-2019<br>N= 164435	Extreme heat	Coercive measures (seclusion, restraints), violent incidents	Patient medical record and patient assessment tool (scale)
Escobar Carias 2022	Indonesia<br>Urban	Cohort	Participants from IFLS and RISE surveys<br>N=NR	Flood	Depression (adults), emotional functioning (children)	Multiple patient assessment tools (score, inventory)
Exenberger 2019	India<br>Urban	Cross-sectional	Mothers and children affected by tsunami<br>N= 244	Tsunami-related floods	Children's PTSD symptoms, parent PTSD symptoms, parent-reported PTSD symptoms for children	Multiple patient assessment tools (scale, parent report)
Florido 2021	Global	Retrospective longitudinal	Suicides<br>N=NR	Heatwave	Suicide deaths	Administrative data
Fontalba-Navas 2017	Spain<br>Semi-urban	Case-control	Adults<br>N=161	Flood	PTSD	Questionnaire
French 2019	England<br>Rural	Cross-sectional	Adults affected by the flood<br>N=590	Flood	Anxiety, depression, health related quality of life, PTSD	Multiple patient assessment tools (questionnaire, checklist, survey)
Gallagher 2019	Australia<br>Rural	Longitudinal	Past and current residents affected by bushfire<br>N=642	Wildfire	Depression, PTSD	Multiple patient assessment tools (checklist, questionnaire)
Gao 2023	Australia<br>NR	Cross-sectional	Adults exposed to smoke<br>N=709	Wildfire	Anxiety, depression, general psychological distress, PTSS, somatic symptoms	Multiple patient assessment tools (questionnaire, scale, survey)
Garfin 2022	USA<br>Both	Cohort	Florida residents<br>N=1113	Hurricane	Functional impairment, generalized worries, global distress, PTSS	Multiple patient assessment tools (screen, scale, inventory)
Grineski 2022	USA<br>Urban	Cross-sectional	Adult Texas residents<br>N=790	Winter storm	PTSD	Survey
Hanigan 2018	Australia<br>Rural	Cross-sectional	Adults<br>N=5312	Drought	Distress	Survey
Hassan 2018	India<br>Rural	Cross-sectional	Child survivors of the floods<br>N=64	Flood	PTSS	Survey and patient assessment tool (scale)

First Author, Year	Country of study Urban/Rural	Study design	Population (N)	Type of extreme weather event	Mental health outcome(s) evaluated	Measure(s) of mental health impact used
He 2022	China<br>Urban	Administrative data review	Patients with schizophrenia<br>N=11005	Cold spell	Schizophrenia admissions	Electronic medical record system
Heatherington 2017/2018	Canada<br>Urban	Cohort	Pregnant and postpartum women<br>N=923	Flood	Anxiety, depression, PTSD	Multiple patient assessment tools (scales, score)
Hooper 2018	Australia<br>Rural	Cross-sectional	Adults affected by bushfire<br>N=65	Wildfire	Coping strategies, PTG, posttraumatic stress	Multiple patient assessment tools (scale, survey, inventory)
Hossain 2021	Bangladesh<br>NR	Cross-sectional	Adult cyclone survivors<br>N= 478	Cyclone	Anxiety, depression, psychological distress, suicidal ideation	Survey
Hu 2020	China<br>NR	Administrative data	Emergency ambulance dispatches from 2013-2017<br>N= NR	Heatwave	Suicide-related ambulance calls	Administrative data
Idris 2018	Malaysia<br>Both	Cross-sectional	Adults >18 years<br>N=602	Flood	PTSD	Questionnaire
Jermacane 2018	UK<br>Suburban	Longitudinal	Residents affected by flood<br>N=988	Flood	Anxiety, depression, PTSD	Multiple patient assessment tools (scale, checklist, questionnaire)
Jing 2022	China<br>Urban	Cross-sectional	Residents who experienced flood<br>N=572	Flood	Depression-anxiety-stress, PTSD	Multiple patient assessment tools (scales)
Kar 2021	India<br>Rural	Cross-sectional	Village residents impacted by cyclone<br>N=80	Cyclone	Anxiety, depression, PTSD	Multiple patient assessment tools (questionnaire, checklist)
Kim 2019	Multiple<br>Both	Time-series	Suicides included in study<br>N= 1320148	Extreme heat	Suicide	Administrative data
Kollanus 2021	Finland<br>Both	Retrospective cohort	Deaths during heatwaves<br>N=NR	Heatwaves	Mental and behavioral disorder related deaths	Administrative data
Kubo 2021	Japan<br>NR	Time-stratified case-crossover	Self-harm cases >10 years<br>N=151801	Extreme heat	Self-harm ambulance cases	Administrative data
Labarda 2018	Philippines<br>Urban	Cohort	Adults in disaster relief training program<br>N=361	Typhoon	General psychological distress, posttraumatic stress	Multiple patient assessment tools (checklist, scale)

First Author, Year	Country of study Urban/Rural	Study design	Population (N)	Type of extreme weather event	Mental health outcome(s) evaluated	Measure(s) of mental health impact used
Labarda 2020	Philippines<br>Both	Cross-sectional	Adult typhoon survivors<br>N=345	Typhoon	Depression-anxiety-stress, PTSD	Multiple patient assessment tools (checklist, scale)
Lai 2021	USA<br>Suburban	Longitudinal	Children from 6-16 years old<br>N=1707	Hurricane	PTSS	Survey
Lai 2024	USA<br>NR	Cohort	Adolescents<br>N=240365	Hurricane	Alcohol use, cannabis use, cigarette use, sadness, suicidal ideation	Survey
Laurito 2022	Indonesia<br>NR	Cohort	Adults<br>N= 12447	Storm surge floods (Tsunami)	Post-Traumatic Stress Reactivity	Patient assessment tool (checklist)
Lavenda 2017	Philippines<br>NR	Cross-sectional	Adults impacted by typhoon<br>N=1001	Typhoon	Acute stress disorder, psychological distress	Multiple patient assessment tools (survey, scale)
Lawton 2023	Indonesia<br>NR	Time-series study	Adult respondents<br>N= 615	Storm surge floods (Tsunami)	Post-Traumatic Stress Reactivity	Patient assessment tool (checklist)
Lee 2018	South Korea<br>Urban	Modelling	Hospital admissions due to mental disease<br>N=166579	Extreme Heat	Emergency admissions due to anxiety, dementia, depression, mental health, schizophrenia	Administrative data
Lee 2019	South Korea<br>Urban	time-stratified case-crossover	People who died by suicide<br>N= 30704	Asian dust storms	Suicide	Death statistics database
Lenane 2019	USA<br>NR	Cohort	Adults >65 years<br>N= 2194	Hurricane	Depressive symptoms, high stress, PTSD	Interview and multiple patient assessment tools (scale, score, checklist)
Li 2021	USA<br>Urban	Cross-sectional	Adults<br>N=272	Hurricane	Hurricane-related distress, PTSD	Multiple patient assessment tools (checklist, scale)
Liang 2023a	China<br>Urban	Cross-sectional	Adults >18 years who experienced flooding in July 2021 followed by a COVID-19 pandemic resurgence<br>N= 977	Flood	Anxiety, PTSS, rumination	Multiple patient assessment tools (survey, scales)
Liang 2023b	Philippines<br>Urban	Longitudinal	Adult survivors<br>N=168	Typhoon	Anxiety, depression, insomnia, stress	Multiple patient assessments (index, scale)
Lieberman-Cribbin 2017	USA<br>urban	Cross-sectional	NYC Residents<br>N=1231	Hurricane, flooding	Depression, GAD, PTSD	Multiple patient assessment tools (checklist, questionnaire)

First Author, Year	Country of study Urban/Rural	Study design	Population (N)	Type of extreme weather event	Mental health outcome(s) evaluated	Measure(s) of mental health impact used
Liu 2018	China<br>Urban	Case-crossover	New hospitalized people with mental health conditions<br>N= 3573	Heatwave	Mental diseases-related hospital admissions	Admissions data
Mamun 2019	Bangladesh<br>Rural	Cross-sectional	Adult females<br>N=111	Cyclone	Depression	Interview
Mamun 2021	Bangladesh<br>Rural	Cross-sectional interview	Adult flood survivors<br>N= 348	Flood	Anxiety, depression, PTSD, suicidal attempts, suicidal ideation, suicidal plan	Interview and multiple patient assessment tools (survey, questionnaire)
Mao 2024	Canada<br>Both	Cross-sectional	Text4Hope respondents<br>N=298	Wildfires	MDD	Survey
Mathew 2021	India<br>Both	Cross-sectional	Adolescents in flood affected areas<br>N= 670	Flood	PTSD	Interview and questionnaire
Matthews 2019	Australia<br>Rural	Cross-sectional	Adults affected by flood<br>N=2180	Flood	Probable anxiety, probable depression, probably PTSD, still distressed, suicidal ideation	Multiple patient assessment tools (checklist, questionnaire, survey)
McCann-Pineo 2021	USA<br>NR	Retrospective analysis from cross-sectional data	Adult hurricane survivors<br>N= 1687	Hurricane	Opioid abuse	Questionnaire
McDonald 2019	USA<br>Both	Longitudinal	Children<br>N=346	Tornado	Pretornado trauma exposure, PTSD	Multiple patient assessment tools (indexes)
McGuire 2018	USA<br>NR	Cross-sectional	Adults exposed to hurricane<br>N= 810	Hurricane	MDD, PTSD	Interview and questionnaire
Meltzer 2020	USA<br>Urban	Cohort	Adolescents<br>N=648	Hurricane	Adolescent psychological distress	Survey
Milojevic 2017	England<br>Both	Quasi-experimental, Interrupted Time series	Primary care practices<br>N=930	Flood	Drugs used in management of common mental disorders	Administrative data
Molyneaux 2020	Australia<br>Both	Cross-sectional	Adults >18 years<br>N=1016	Wildfire	Alcohol use, depression, PTSD	Multiple patient assessment tools (checklist, questionnaire, test)
Moosavi 2019	Canada<br>Urban	Cross-sectional	Residents of Fort McMurray >18 years<br>N=290	Wildfire	GAD, MDD, PTSD, substance abuse	Multiple patient assessment tools (checklist, questionnaire, test)

First Author, Year	Country of study Urban/Rural	Study design	Population (N)	Type of extreme weather event	Mental health outcome(s) evaluated	Measure(s) of mental health impact used
Mordeno 2017a	Philippines<br>Rural	Cross-sectional	Village chiefs<br>N=523	Typhoon	GAD, PTSD	Multiple patient assessment tools (checklist, survey)
Mordeno 2017b	Philippines<br>Rural	Cross-sectional	Young adults<br>N=632	Typhoon	MDD, PTSD	Multiple patient assessment tools (checklist, questionnaire)
Mordeno 2018	Philippines<br>Urban	Cross-sectional	Child and adolescent survivors of typhoon<br>N=225	Typhoon	Acute stress disorder, centrality of event and mental health outcomes, depression, sensory-based memory quality of traumatic event	Multiple patient assessment tools (interview, scale, questionnaire)
Mulchandani 2020	UK<br>NR	Cohort	People unaffected, disrupted, or flooded<br>N=819	Flood	Anxiety, depression, PTSD	Multiple patient assessment tools (checklist, questionnaire)
Munro 2017	UK<br>Urban	Cross-sectional	People affected by the flood<br>N=622	Flood	Anxiety, depression, PTSD	Multiple patient assessment tools (checklist, questionnaire, survey)
Nalipay 2018	Philippines<br>Urban	Cross-sectional	College students survivors of Typhoon<br>N=446	Typhoon	Positive metacognitions and positive meta-emotions, posttraumatic cognitions inventory, PTG, PTSD	Multiple patient assessment tools (checklist, inventory, questionnaire)
Nasri 2019	Indonesia<br>NR	Cross-sectional	Flood victims<br>N= 356	Flood	PTSD	Questionnaire
Nicosia 2022	USA<br>Urban	Convenience sampling from a movement trial	Adults with cognitive impairment and caregivers<br>N= 13	Wildfire	Perceived stress	Survey
Niu 2020	China<br>Urban	Time-series	Emergency visits for mental and behavioral disorders<br>N=16606	Extreme temperatures	ED visits for mental health related illness, mood disorders, neurotic disorders, psychoactive substance use, schizophrenia	Administrative data
Nori-Sarma 2022	USA<br>NR	Case-crossover	ED visits and health insurance claims for adults >18 years<br>N=3496762	Extreme heat	ED visits relating to adult personality and behaviour disorders; anxiety, stress-related and somatoform disorders; childhood-onset behavioural disorders; mental health; miscellaneous mental health visits; schizophrenia, schizotypal, and delusional disorders; self-harm; substance use	ED visit claims
Obradovich 2018	USA<br>Both	Cross-sectional	US Residents<br>N=1961743	Hurricane	Self-reported mental health difficulties in past 30 days	Administrative data
Pailler 2018	India<br>Both	Cohort	Adults aged 18-60<br>N= 16227	Drought	Depressive symptoms	Survey

First Author, Year	Country of study Urban/Rural	Study design	Population (N)	Type of extreme weather event	Mental health outcome(s) evaluated	Measure(s) of mental health impact used
Pan 2022	Japan<br>NR	Time-series	Cause of death records<br>N=2416707	Extreme heat	Suicide deaths	Death certificate data
Peng 2017	China<br>Urban	Time-series	Mental disorder hospital admissions<br>N=93971	Extreme temperatures	All mental disorder hospital admissions	Administrative data
Philip 2023	India<br>NR	Mixed methods	Adolescents aged 12-18<br>N= 100	Flood	Anxiety, depression, impact of event, stress	Interview and multiple patient assessment tools (scales)
Phillippi 2019	USA<br>Urban	Time series	Adults<br>N=22196	Flood	Service use for anxiety disorders, bipolar disorders, depressive disorders, schizophrenic and psychotic disorders, substance and addictive disorders, all other mental health/behaviour disorders	Medicaid claims data
Psarros 2017	Greece<br>Rural	Cross-sectional	Disaster-stricken residents<br>N=92	Wildfire	Anxiety, fear of death, insomnia, PTSD, psychopathology symptoms	Multiple patient assessment tools (scale, self-report, inventory, checklist)
Quan 2017	China<br>Urban	Cross-sectional	Adolescents<br>N=951	Rainstorm	PTSD, resilience, rumination	Multiple patient assessment tools (checklist, scales)
Rahman 2023	Bangladesh<br>Rural	Cross-sectional	Women exposed to flood<br>N=393	Flood	Anxiety, depression, stress	Survey
Raker 2019	USA<br>Urban	Cohort	Low-income mothers<br>N=468	Hurricane	Psychological distress, PTSS	Multiple patient assessment tools (scales)
Richardson 2020	India<br>Rural	Cohort	Suicides in adults >15 years<br>N-9456	Flood, drought	Suicide deaths	Administrative data
Ritchie 2020/2021	Canada<br>Urban	Cross-sectional	College students exposed to fire<br>N-329	Wildfire	Drug-related problems, GAD, MDD, problem alcohol use, PTSD	Multiple patient assessment tools (checklist, questionnaire, tests)
Schultz 2020	Bahamas<br>Urban	Qualitative	Clinic staff and disaster survivors<br>N=NR	Hurricane	Acute psychological distress	Observation
Schwartz 2017	USA<br>Urban	Longitudinal	Adults<br>N=130	Hurricane	Anxiety, depression, problem alcohol use, PTSD	Multiple patient assessment tools (questionnaire, survey)
Schwartz 2019	USA<br>Urban	Cohort	Adults<br>N=2767	Hurricane	PTSD	Survey

First Author, Year	Country of study Urban/Rural	Study design	Population (N)	Type of extreme weather event	Mental health outcome(s) evaluated	Measure(s) of mental health impact used
Shannonhouse 2019	Botswana<br>Rural	Cohort	Undergraduate students<br>N=300	Drought	Trauma symptoms	Questionnaire
Sherbakov 2018	USA<br>Both	Retrospective longitudinal	Hospitalizations<br>N=6820027	Heatwave	Hospitalizations	Admissions data
Shih 2021	Taiwan<br>Both	Observational, prospective population-based	Adults<br>N=897689	Typhoon	Monthly visits for adjustment reactions, anxiety, depressive disorders, episodic mood disorders, PTSD, stress-associated insomnia, all stress-associated illness	National Health Insurance data
Silveira 2021	USA<br>Urban	Cross-sectional	Adults aged 18-64 exposed to fire<br>N=725	Wildfire	GAD, MDD, PTSD	Multiple patient assessment tools (checklists, questionnaire)
Sirey 2017	USA<br>Urban	Cross-sectional	Adults >60 years in flood zones<br>N=1512	Hurricane	Anxiety, depression, PTSD, suicidal ideation	Interview and multiple patient assessment tools (assessment, checklist, questionnaire)
Sonpaveerawong 2019	Thailand<br>Rural	Cross-sectional	Adult flood survivors<br>N=326	Flood	Alcohol misuse, depression, psychological distress, PTSD, risk of suicide, thoughts of suicide	Survey
Sugg 2019	USA<br>Urban	Program review analysis	Adolescents and young adults<br>N=59000	Heat	Crisis help seeking	Administrative data
Sun 2021	USA<br>Both	Time-stratified	Adults >18 years old<br>N=21996670	Extreme heat	Mental health disorder ED visits	Administrative data
Tempest 2017	UK<br>NR	Cross-sectional	Adults<br>N=2006	Flood	Anxiety, depression, PTSD	Multiple patient assessment tools (checklist, questionnaires)
Thomas 2021	India<br>Rural	Cross-sectional	Adults aged 15-65<br>N=171	Flood	Anger, mania, problems with memory, psychosis, sleep disorders, somatic symptoms, substance abuse, suicidal ideation, symptoms of anxiety, symptoms of depression, symptoms of dissociation	Survey
Tran 2023	Vietnam<br>NR	Time-series	Hospitalization admissions<br>N=NR	Extreme heat	Mental health admissions	Administrative admissions data
Verstraeten 2020	Canada<br>Both	Longitudinal	Women exposed to wildfire<br>N=200	Wildfire	Perinatal PTSD, peritraumatic dissociative experiences, peritraumatic distress	Multiple patient assessment tools (scale, inventory, questionnaire)
Viswanthan 2019	India<br>Rural	Cross-sectional	Farmers<br>N=194	Drought	Depression, suicidal ideation	Questionnaire

First Author, Year	Country of study Urban/Rural	Study design	Population (N)	Type of extreme weather event	Mental health outcome(s) evaluated	Measure(s) of mental health impact used
Waite 2017	UK<br>NR	Cross-sectional	Adults >18<br>N=2126	Flood	Anxiety, depression, PTSD	Multiple patient assessment tools (questionnaire, checklist)
Wang 2023	China<br>Urban	Cross-sectional	Residents of Zhengzhou	Flood	Anxiety, depression, PTSD, stress	Multiple patient assessment tools (checklist, scales)
Wei 2020	China<br>Urban	Modelling	Schizophrenia admissions<br>N=36607	Flood	Schizophrenia admissions	Administrative data
Wettstein 2024	USA<br>Urban	Cohort	Adults<br>N=7115690	Wildfire	Psychotropic medication prescription rates for antidepressants, antipsychotics, anxiolytics, hypnotics, mood stabilizers	Administrative data
Wheeler 2018	Australia<br>Rural	Cross-sectional	Irrigator farmers<br>N=1000	Drought	Psychological distress	Survey
Wondmagegn 2021	Australia<br>Urban	Modelling	Heatwave associated hospitalizations<br>N=1161	Heatwave	Mental health related ED visits	Administrative admissions data
Wu 2022	China<br>Both	Modelling	Schizophrenia patients<br>N=14056	Extreme precipitation	Schizophrenia hospitalizations	Medical record data
Xu 2018a	China<br>Urban	Cross-sectional	Adolescents<br>N=247	Tornado	PTSD	Survey
Xu 2019	Australia<br>Both	Modelling	Emergency department visits<br>N=NR	Heatwave	Mental and behavioural disorder ER visits	Administrative data
Xu 2019b	Australia<br>Urban	Retrospective cohort	Hospitalizations for Alzheimer's disease<br>N=907	Heatwaves	Alzheimer's hospitalizations, Alzheimer's deaths within 2 months of hospitalization	Administrative data
Yoo 2021a	USA<br>Urban	Modelling	ED visits due to mental-health related illnesses<br>N=94636	Extreme temperatures	ED visits for mental health related illness	Administrative data
Yoo 2021b	USA<br>Urban	Modelling	ED visits due to mental health-related illnesses<br>N=2893794	Extreme heat	Anxiety disorder ED visits, dementia ED vistis, mental disorder ED vistis, mood disorder ED visits, psychoactive substance use ED visits, schizophrenia ED vistis	Administrative data
Yuan 2018a	China<br>Urban	Cross-sectional	Adolescents<br>N=431	Tornado	Burnout, dispositional mindfulness, PTSD, regulatory emotional self-efficacy	Multiple patient assessment tools (scales, questionnaires)

First Author, Year	Country of study Urban/Rural	Study design	Population (N)	Type of extreme weather event	Mental health outcome(s) evaluated	Measure(s) of mental health impact used
Zeligman 2020	Botswana<br>Both	Cross-sectional	University students<br>N=300	Drought	Posttraumatic growth, trauma symptoms	Multiple patient assessment tools (inventory, questionnaire)
Zhang 2020	China<br>Urban	Case-crossover	Outpatient visits<br>N=1133220	Extreme temperatures	Outpatient visits relating to affective disorders, anxiety, depressive, organic mental disorders, schizophrenia	Administrative data
Zhen 2018	China<br>Urban	Cross-sectional	Adults<br>N=187	Flood	Depression, Posttraumatic negative cognition	Multiple patient assessment tools (inventory, scale)
Zhou 2023	China<br>Both	Case-control	Suicides<br>N=432008	Extreme temperatures	Death by suicide	Administrative data

‡ED: Emergency Department; GAD: generalized anxiety disorder; MDD: major depressive disorder; OCD: obsessive-compulsive disorder; PTG: post-traumatic growth; PTSD: post-traumatic stress disorder; PTSS: post-traumatic stress symptoms;
