# Supplementary material for: Mental health outcomes relating to climate-sensitive extreme weather events: A scoping review of measures of mental health impact
Source: J Clim Chang Health. 2026 Sep-Oct;31:100743. doi: 10.1016/j.joclim.2026.100743 (PMC13573736; doi:10.1016/j.joclim.2026.100743)
Supplement: Supplementary file 3 [file mmc3.pdf]

Supplementary Material 3 - Table S2. Categorization based on the groupings of diagnostic criteria and codes from the Diagnostic and Statistical Manual of Mental Disorders, Fifth Edition (DSM-5) and the International Classification of Diseases 10th and 11th revision and primary and secondary sources of measures of mental health impact by mental health outcome

ANXIETY DISORDERS			
**Outcome Measure**	**Measures of Mental Health Impact**		**ICD Codes**
	**Primary Sources**	**Secondary Sources**	
Agoraphobia	Mini International Neuropsychiatric Interview (MINI)		
Anxiety	Beck's Anxiety Inventory (BAI)<br>Brief symptom rating scale (BSRS-5)<br>Depression Anxiety and Stress Scales (DASS-21) [4]<br>Focus groups and interviews<br>General Anxiety Disorder - 7 (GAD-7) [6]<br>Generalized Anxiety Disorder - 2 (GAD-2) [6]<br>Hospital Anxiety and Depression Scale (HADS) [2]<br>Mental health and wellbeing survey<br>Patient Health Questionnaire-4 (PHQ-4)<br>Self-described through photovoice<br>Self-rating Anxiety Scale (SAS)<br>Spielberger State Anxiety Scale (SSA)<br>State-Trait Anxiety Inventory (STAI)		
Anxiety disorder ED visits		ICD 9 and 10	ICD9: 298.2, 300, 306, 307.8, 308, 309, 310.8; ICD10: F40-49
Anxiety emergency admissions		ICD 10	F40, F41
Anxiety outpatient visits		ICD-10	F40-F41
Anxiety-related ED visits and hospitalization		ICD-9 codes	
Anxiety, stress-related and somatoform disorder ED visits		Clinical Classification Software codes: 650, 651	
Depression, anxiety, and stress	Depression Anxiety Stress Scales (DASS-21) [3]		
Generalized Anxiety Disorder (GAD)	General Anxiety Disorder - 7 (GAD-7) [6]<br>Mini International Neuropsychiatric Interview (MINI)<br>Patient Health Questionnaire-4 (PHQ-4)		
Monthly visits for anxiety		ICD 9, CM (Clinical modification)	300.0, 300.2, 300.3, 308.3
Panic Disorder	Mini International Neuropsychiatric Interview (MINI)		
Probable Anxiety	Generalized Anxiety Disorder scale (GAD-2)		
Psychotropic medication prescription rates - anxiolytics		MarketScan data during fire and pre-fire periods	
Service use for anxiety disorders		ICD 10	F40-F419
Social phobia	Mini International Neuropsychiatric Interview (MINI)		
Symptoms of Anxiety	DSM-5 criteria, grading of 1-4; a rating of 2 or higher was presence of the symptom		
**BIPOLAR AND RELATED DISORDERS**			
**Outcome Measure**	**Measures of Mental Health Impact**		**ICD Codes**
	**Primary Sources**	**Secondary Sources**	
Service use for bipolar disorders		ICD 10	F30-F31, F34-F39
**DEPRESSIVE DISORDERS**			
**Outcome Measure**	**Measures of Mental Health Impact**		**ICD Codes**
	**Primary Sources**	**Secondary Sources**	
Depression	Beck's Depression Inventory (BDI)<br>Brief symptom rating scale (BSRS-5)<br>Center for Epidemiologic Studies Depression Scale for Children (CES-DC) [2]<br>Center for Epidemiological Studies Depression Score (CES-D) [3]<br>Depression Anxiety and Stress Scales (DASS-21) [4]<br>Depression Self-Rating Scale for Children		

	Focus groups and interviews<br>General Health Questionnaire Twelve Plus R (GHQ-12-Plus-R) - DS8 at end<br>Hospital Anxiety and Depression Scale<br>Mental health and wellbeing survey<br>Patient Health Questionnaire - 9 (PHQ-9) [15]<br>Patient Health Questionnaire-4 (PHQ-4) [2]<br>Patient Health Questionnaire -2 (PHQ-2) [6]<br>Patient Health Questionnaire – adolescents (PHQ-A)<br>Self report of having been told they have depression, major depression, dysthymia, or minor depression		
Depression emergency admissions		ICD 10	
Depression, anxiety, and stress	Depression Anxiety Stress Scales (DASS-21) [3]		F32, F33
Depressive disorder outpatient visits		ICD-10	
Depressive symptoms	Center for Epidemiological Studies Depression Score (CESD-10)<br>Survey questions about sadness, interest, and energy<br>DSM-5 criteria, grading of 1-4; a rating of 2 or higher was presence of the symptom		
Major Depressive Disorder (MDD)	Mini International Neuropsychiatric Interview (MINI)<br>Patient Health Questionnaire (PHQ) 9 [7]<br>Patient Health Questionnaire (PHQ)		
Major depressive episodes	Patient Health Questionnaire (PHQ) 9 [2]		
Monthly visits for depressive disorders		ICD 9, CM (Clinical modification)	
Probable Depression	Patient Health Questionnaire (PHQ-2)		296.2-296.3, 300.4, 311
Psychotropic medication prescription rates - antidepressants		MarketScan data during fire and pre-fire periods	
Service use for depressive disorders		ICD 10	F32-F33
**DISSOCIATIVE DISORDERS**			
**Outcome Measure**	**Measures of Mental Health Impact**		**ICD Codes**
	**Primary Sources**	**Secondary Sources**	
Peritraumatic dissociative experiences	Peritraumatic Dissociative Experiences Questionnaire (PDEQ)		
Symptoms of dissociation	DSM-5 criteria: grading of 1-4; a rating of 2 or higher was presence of the symptom		
**INTELLECTUAL DISABILITY**			
**Outcome Measure**	**Measures of Mental Health Impact**		**ICD Codes**
	**Primary Sources**	**Secondary Sources**	
Admissions for mental retardation		ICD 10 [2]	F70-F79
**GENERAL MENTAL HEALTH OUTCOMES**			
**Outcome Measure**	**Measures of Mental Health Impact**		**ICD Codes**
	**Primary Sources**	**Secondary Sources**	
Centrality of Event and Mental Health Outcomes	Centrality of Event Scale (CES)		
Complex behavioural disorders - admissions		ICD 10	F50-F59
Days a month of poor physical or mental health	Survey with question about days of poor mental health in previous month		
General psychiatric morbidity	General Health Questionnaire-28 (GHQ-28)		
Health Related Quality of Life	EQ-5D-5L		
Mental and behavioral disorders - admissions		ICD 10	F00-F99
Mental and behavioral disorders – ER visits		ICD 10	F00-F99
Mental and behavioral disorder related deaths		ICD 10	F00-F99
Mental disorder - admissions		ICD 10 [6]	F00-F99
Mental disorder – ED visits		ICD 10<br>ICD 9	ICD 9: 290-319; ICD 10: F00-F99
Mental disorder outpatient visits		ICD-10	F00-F09

Mental health admissions		ICD-10 [4]<br>ICD 9	ICD 10: F00-F99<br>ICD 9: 290-319
Mental health ED visits		Clinical Classification Software codes: 650-652, 654, 655, 657-662, 670 [2]<br>ICD 10 [5]<br>ICD 9 [3]	F00-F99.9<br>ICD 9: 290-319
Mental health emergency admissions		ICD 10	F00-F99
Mental health related ED visit and hospitalizations		ICD-9 codes	ICD9 290-299, 300, 301, 302-316, 980, E950-E959
Mental health service utilisation	Perceived Need for Care Questionnaire (PHCQ)<br>ICD10		
Poor mental health days	BRFSS 2004-2006 surveys (Behavioral Risk Factor Surveillance System)		
Psychiatric emergency visits		Administrative data	
Psychopathology symptoms	Symptom Checklist-90-R (SCL-90-R)		
Self-reported mental health difficulties in past 30 days	Behavioral Risk Factor Surveillance System (BRFSS)		
Severe Mental Illness	Kessler Psychological Distress Scale (K6)		
Service use for all other mental health/behavioral disorders		ICD-10	
**MOOD DISORDERS**			
**Outcome Measure**	**Measures of Mental Health Impact**		**ICD Codes**
	**Primary Sources**	**Secondary Sources**	
Affective disorder outpatient visits (minus depression)		ICD 10	F30-F31, F34-F39
Behavior and mood disorders usually associated with childhood and youth - admissions		ICD 10	F90-F98
Mania	DSM-5 criteria, grading of 1-4; a rating of 2 or higher was presence of the symptom		
Monthly visits for episodic mood disorders		ICD 9, CM (Clinical modification)	296
Mood (affective) disorders		ICD-10 codes	F30-F39
Mood disorder ED visits		Clinical Classification Software Codes: 657<br>ICD 10 [2]<br>ICD 9 [2]	ICD10: F30-39<br>ICD9: 296, 298, 300.4, 311
Mood disorder ED visits and hospitalisations		ICD-9 codes	
Mood disorder hospitalizations		ICD 10	F30-F39
Neurotic disorder ED visits		ICD 10	F40-F49
Neurotic disorder hospitalizations		ICD 10	F40-F59
Neurotic, stress-related and somatoform disorder admissions		ICD 10	F40-F49
Psychotropic medication prescription rates - mood stabilizers		MarketScan data during fire and pre-fire periods	
**NEUROCOGNITIVE DISORDERS**			
**Outcome Measure**	**Measures of Mental Health Impact**		**ICD Codes**
	**Primary Sources**	**Secondary Sources**	
Alzheimer's deaths within 2 months of hospitalization		Registrar General Death Database records	
Alzheimer's disease urgent hospital admissions		ICD 9	331
Alzheimer's hospitalizations		Database for admitted patients	
Dementia ED visits		ICD 9 and 10	ICD9: 290, 294.1, 294.2;<br>ICD10: F00-F03
Dementia emergency admissions		ICD 10	F00-F03, G30, G31
Dysfunctional cognitions	Cognitive Distortion Scales (CDS)		

NEURODEVELOPMENTAL DISORDERS			
**Outcome Measure**	**Measures of Mental Health Impact**		**ICD Codes**
	**Primary Sources**	**Secondary Sources**	
Developmental disorder admissions		ICD10 [3]	F70-F79<br>F80-F98
**OBSESSIVE-COMPULSIVE AND RELATED DISORDERS**			
**Outcome Measure**	**Measures of Mental Health Impact**		**ICD Codes**
	**Primary Sources**	**Secondary Sources**	
Obsessive Compulsive Disorder (OCD)	Mini International Neuropsychiatric Interview (MINI)		
**PERSONALITY DISORDERS**			
**Outcome Measure**	**Measures of Mental Health Impact**		**ICD Codes**
	**Primary Sources**	**Secondary Sources**	
Adult personality and behavior disorder admissions		ICD 10 [3]	F60-F69
Adult personality and behavior disorder ED visits		Clinical Classification Software codes: 658	
**SCHIZOPHRENIA SPECTRUM AND OTHER PSYCHOTIC DISORDERS**			
**Outcome Measure**	**Measures of Mental Health Impact**		**ICD Codes**
	**Primary Sources**	**Secondary Sources**	
Psychosis	DSM-5 criteria, grading of 1-4; a rating of 1 or higher was presence of the symptom		
Psychosis ED visits and hospitalizations		ICD-9 codes	ICD9 290-299
Psychotropic medication prescription rates - antipsychotics		MarketScan data during fire and pre-fire periods	
Psychotropic medication prescription rates - hypnotics		MarketScan data during fire and pre-fire periods	
Schizophrenia admissions		ICD-10 [5]	F20.0–20.9, F20-F29
Schizophrenia ED visits		ICD-10 [2]<br>ICD-9	ICD9: 295, 297, 298.1-298.9, 301.2; ICD10: F20-F29
Schizophrenia emergency admissions		ICD-10	F20, F21
Schizophrenia outpatient visits		ICD-10	F20-F29
Schizophrenia, schizotypal and delusional disorder ED visits		Clinical Classification Software codes: 659	
Schizophrenia, schizotypal and delusional disorders		ICD-10	F20-F29
Service use for schizophrenic and psychotic disorders		ICD-10	F20-F29
**SLEEP-WAKE DISORDERS**			
**Outcome Measure**	**Measures of Mental Health Impact**		**ICD Codes**
	**Primary Sources**	**Secondary Sources**	
Insomnia	Athens Insomnia Scale (AIS)<br>Clinician Administered PTSD Scale item E6<br>Insomnia Severity Index (ISI) [2]<br>Pittsburgh Sleep Quality Index (PSQI, PSQI-A)		
Monthly visits for stress-associated insomnia		ICD 9, CM (Clinical modification)	780.5, 307.4
Sleep disorders	DSM-5 criteria, grading of 1-4; a rating of 2 or higher was presence of the symptom		
**SOMATIC SYMPTOM AND RELATED DISORDERS**			
**Outcome Measure**	**Measures of Mental Health Impact**		**ICD Codes**
	**Primary Sources**	**Secondary Sources**	
Anxiety, stress-related and somatoform disorder ED visits		Clinical Classification Software codes: 650, 651	
Complex behavioural disorders associated with physiological disorders and somatic factors admissions		ICD-10	F50-F59

Neurotic, stress-related and somatoform disorder admissions		ICD-10 [2]	F40-F49
Somatic symptoms	PHQ-15<br>DSM-5 criteria, grading of 1-4; a rating of 2 or higher was presence of the symptom		
**SUBSTANCE-RELATED AND ADDICTIVE DISORDERS**			
**Outcome Measure**	**Measures of Mental Health Impact**		**ICD Codes**
	**Primary Sources**	**Secondary Sources**	
Alcohol and substance use ED visits and hospitalizations		ICD-9	ICD9 980
Alcohol use	Consumption Scale of the Alcohol Use Disorder Identification Test (AUDIT-C)<br>Survey, dichotomous use question		
Alcohol use disorder	Mini International Neuropsychiatric Interview (MINI)		
Binge drinking in past year	Heavy alcohol consumption by self-report		
Cannabis use	Survey, dichotomous use question		
Cigarette use	Survey, dichotomous use question		
Drug use disorder	Mini International Neuropsychiatric Interview (MINI)		
Drug-related problems	Drug Use Disorder Identification Test (DUDIT)		
Drugs Used in Management of Common Mental Disorders	Change of the number of antidepressant drug items prescribed at primary care practices		
Mental and behavioral disorders caused by use of psychoactive substances - admissions		ICD-10 [2]	F10-F19
Nicotine dependence	Fagerstrom Test for Nicotine Dependence		
Opioid abuse	Opioid Risk Tool (ORT)		
Psychoactive substance use - ED visits		ICD 10 [2]<br>ICD 9	ICD9: 291,292, 303-305;<br>ICD10: F10-F19
Psychoactive substance use - hospitalizations		ICD 10	F10-F19
Recreational drug use	Survey		
Service use for substance and addictive disorders		ICD 10	F10-F19
Substance abuse	Alcohol Use Disorder Identification Test (AUDIT)<br>CRAFFT Questionnaire<br>DSM-5 criteria, grading of 1-4; a rating of 1 or higher was presence of the symptom<br>Drug Use Disorder Identification Test (DUDIT) [2]<br>Self-report		
Substance abuse - Alcohol	Alcohol Use Disorder Identification Test (AUDIT) [2]<br>Alcohol Use Disorder Identification Test- Consumption (AUDIT-C) [2]<br>CRAFFT Questionnaire<br>General Health Questionnaire Twelve Plus R (GHQ-12-Plus-R) - 5 items<br>Survey		
Substance abuse - Drugs	Drug Use Disorder Identification Test (DUDIT)		
Substance use ED visits		Clinical Classification Software codes: 660, 661	
Tobacco use	Tobacco Use Questionnaire		
**SUICIDE AND SELF HARM BEHAVIOR**			
**Outcome Measure**	**Measures of Mental Health Impact**		**ICD Codes**
	**Primary Sources**	**Secondary Sources**	
Crisis help seeking	Crisis line data		
Risk of suicide	General Health Questionnaire Twelve Plus R (GHQ-12-Plus-R) - DS8 at end		
Self-harm	ALSPAC Life of a 16+ teenager questionnaire		
Self-harm ambulance cases		Administrative data	
Self-harm ED visits		Clinical Classification Software codes: 662	
Suicidal Ideation	Brief symptom rating scale (BSRS-5)<br>DSM-5 criteria, grading of 1-4; a rating of 1 or higher was presence of the symptom<br>General Health Questionnaire Twelve Plus R (GHQ-12-Plus-R) - Item 21<br>Interview questions [2]		

	National survey of mental health and wellbeing<br>Patient Health Questionnaire-9 (PHQ-9) final item [2]<br>Patient Health Questionnaire - Adolescent version (PHQ-A)<br>Screening Tool for Assessing Risk of Suicide<br>Suicide Risk Assessment<br>Survey, question about seriously considering attempting suicide		
Suicidal tendency	Mini International Neuropsychiatric Interview (MINI)		
Suicide and intentional harm ED visits and hospitalizations		ICD-9 codes	ICD9 E950-E959
Suicide attempts	Interview<br>National survey of mental health and wellbeing		
Suicide deaths		ICD 10 [7]<br>ICD 9 [3]<br>ICD 8 [1]	ICD 10 X60-X84.9; Y10-Y10.3, Y10-Y34 and Y87.0; ICD 8, 9: E950.0-E958.9; ICD 8: 1950-1957
Suicide watch incidents		Database	
Suicide-related ambulance calls		ICD 10	X60-X84
Suicides per 100,000		ICD 10<br>National database	X60-X84
Thoughts of self-harm	ALSPAC Life of a 16+ teenager questionnaire		
**TRAUMA- AND STRESSOR-RELATED DISORDERS**			
**Outcome Measure**	**Measures of Mental Health Impact**		**ICD Codes**
	**Primary Sources**	**Secondary Sources**	
Acute psychological distress	Clinical staff observation		
Acute stress disorder	Acute Stress Disorder Interview (ASDI)<br>DSM-5 Acute stress disorder symptoms		
Adjustment disorder ED visits and hospitalizations		ICD 9	
Adolescent psychological distress	Caregiver reports		
Anxiety, stress-related and somatoform disorder ED visits		Clinical Classification Software codes: 650, 651	
Avoidant attachment style	Experiences in close relationships scale (ECR)		
Children's PTSD symptoms	Children-s Revised Impact of event scale - 13 (CRIES-13)		
Chronic PTSD	PTSD Checklist - Civilian Version (PCL-C)		
Chronic stress	Self-described through photovoice		
Community drought-related stress	Self report – 6 item Likert scale		
Depression, anxiety, and stress	Depression Anxiety Stress Scales (DASS-21) [3]		
Distress	Kessler Psychological Distress Scale (K10)		
Fire-related PTSD	Posttraumatic Stress Disorder Checklist (PCL-S)		
Global distress	Brief symptom inventory - 18		
High Stress	Perceived Stress Scale		
Hurricane-related distress	Impact of Event Scale (IES-R)		
Monthly visits for adjustment reactions		ICD 9, CM (Clinical modification)	309
Monthly visits for all stress-associated illness		ICD 9, CM (Clinical modification)	
Monthly visits for PTSD		ICD 9, CM (Clinical modification)	309.81
Neurotic, stress-related and somatoform disorder admissions		ICD 10 [2]	F40-F49
Occupational psychosocial stress ("job strain")	Job Content Questionnaire (JCQ)		
Parent PTSD symptoms	Impact of Event Scale - Revised (IES-R)		
Parent reported PTSD symptoms for children	Parent report of the child's reaction to stress (PRCRS)		
Parental Attachment	Inventory of Parent and Peer Attachment (IPPA-R)		
Perceived stress	Perceived stress scale (PSS) [2]		
Perinatal PTSD	Impact of Event Scale - Revised (IES-R)		
Peritraumatic distress	Peritraumatic Distress Inventory (PDI)		
Personal drought-related stress	Self report; 6 item Likert scale		

Positive metacognitions and positive meta-emotions	The Positive Metacognitions and Positive Meta-Emotions Questionnaire (PMCEQ)		
Post Traumatic Stress Reactivity	Posttraumatic Stress Disorder Checklist - Civilian version (PCL-C) [3]		
Posttraumatic Cognitions	Posttraumatic Cognitions Inventory (PTCI) [3]		
Posttraumatic Stress	Impact of Events Scale (IES-R) [2]<br>Posttraumatic Stress Checklist for DSM-5 (PCL-5)<br>PTSD Checklist - Specific (PCL-S)		
Posttraumatic Stress Symptoms (PTSS)	Child Posttraumatic Stress Symptom Scale (PTSS)<br>Children's Revised Impact of Event Scale (CRIES)-8<br>Impact of Events Scale (IES-R)<br>University of California, Los Angeles, Posttraumatic Stress Disorder Reaction Index (UCLA PTSD-RI)		
Probable PTSD	Post Traumatic Stress Disorder Checklist (PCL-6)		
Problems with memory	DSM-5 criteria, grading of 1-4; a rating of 2 or higher was presence of the symptom		
Psychological distress	Brief symptom rating scale (BSRS-5)<br>General Health Questionnaire Twelve Plus R (GHQ-12-Plus-R) - Items 1-12<br>Kessler Psychological Distress Scale (K6) [7]<br>Kessler Psychological Distress Scale (K10) [3]		
PTSD	Child PTSD Symptom Scale (CPSS) [5]<br>Civilian PTSD Questionnaire - Hurricane Sandy specific (PTSD/PCL-S)<br>Clinician-Administered PTSD Scale (CAPS) [2]<br>Composite International Diagnostic Interview (CIDI)<br>General Health Questionnaire Twelve Plus R (GHQ-12-Plus-R) - Items 13-20<br>Impact of Event Scale - Revised (IES-R) [5]<br>PTSD Checklist for DSM5 (PCL 5) [16]<br>Primary Care Posttraumatic Stress Disorder Screen for the DSM-IV modified using a 5 point scale<br>PTSD Checklist (PCL−6) [8]<br>PTSD Checklist (PCL-4)<br>PTSD Checklist - Civilian Version (PCL-C) [5]<br>PTSD Checklist - Specific (PCL-S) [7]<br>Posttraumatic Stress Disorder Checklist Screen (PCL-C)<br>Posttraumatic Stress Disorder Checklist (PCL) [2]<br>Posttraumatic Stress Diagnosis Scale (PDS)<br>PTSD Symptom Scale - Interview (PSS-I)<br>Primary care PTSD screen for DSM 5 (PC-PTSD-5)<br>Single-screening items from DSM-V<br>Trauma Screening Questionnaire (TSQ-10) [2]<br>Trauma Screening Questionnaire (TSQ)<br>Trauma Questionnaire (TQ)	ICD 10	
PTSD Reaction Index	PTSD Reaction Index (PTSD- RI)		
Sensory-Based Memory Quality of Traumatic Event	Trauma Memory Quality Questionnaire (TMQQ)		
Service use for trauma and stress disorders		ICD 10	F43-F439
Still distressed	Brief Weather Disaster Trauma Exposure and Impact Screen [2]		
Stress	Depression Anxiety and Stress Scales (DASS-21) [4]<br>Qualitative interview<br>Self report; 6 items on a Likert Scale		
Trauma symptoms	Harvard Trauma Questionnaire (HTQ) [2]		
**FACTORS INFLUENCING HEALTH STATUS OR CONTACT WITH HEALTH SERVICES**			
**Outcome Measure**	**Measures of Mental Health Impact**		**ICD Codes**
	**Primary Sources**	**Secondary Sources**	
Anger	Dimensions of Anger Reactions Scale-5 (DAR-5)<br>DSM-5 criteria, grading of 1-4; a rating of 2 or higher was presence of the symptom		
Burnout	The Learning Burnout Questionnaire (LBQ) [2]		
Coping Strategies	Brief Coping Orientations to Problems Experienced (COPE)		
Emotional functioning - children	Pediatric Quality of Life Inventory (PedsQL)		
Emotional upheaval	Interview		
Fear of Death	Self report; 5 point Likert scale		

Functional impairment	4 items from 36-item SF Health Survey		
Generalized worries	8 survey items from previous research		
Grief	Interview		
Impact of event	Impact of Event Scale - Revised (IES-R)		
Mental and Emotional Well-Being	Interview		
Mindfulness	Mindfulness Attention Awareness Scale (MAAS) [3]		
Posttraumatic Growth (PTG)	Posttraumatic Growth Inventory (PTGI) [3]<br>Post-Traumatic Growth Inventory (PTGI-R)		
Physical Activity and Well-Being	Interview<br>Survey		
Regulatory emotional self-efficacy	Regulatory emotional self-efficacy questionnaire (RESEQ)		
Resilience	Connor & Davidson Resilience Scale (CD-RISC)		
Rumination	Abbreviated Ruminative Responses Scale<br>Subscale of cognitive emotion regulation questionnaire		
Sadness	Survey		
Well-being	Survey<br>World Health Organization - Five Well-Being Scale (WHO-5)		
