# Supplementary material for: Mental health outcomes relating to climate-sensitive extreme weather events: A scoping review of measures of mental health impact
Source: J Clim Chang Health. 2026 Sep-Oct;31:100743. doi: 10.1016/j.joclim.2026.100743 (PMC13573736; doi:10.1016/j.joclim.2026.100743)
Supplement: Supplementary file 4 [file mmc4.pdf]

Supplementary Material 4 - Table S3

Frequency of mental health outcome measures for type of extreme weather event

EWE	# of papers	Rural/urban (frequency)	Countries (frequency)	Mental Health Outcome Measures (frequency)
Cyclone	3	Rural (2)	Bangladesh (2), India	Anxiety (2)<br>Depression (3)<br>Psychological distress<br>Posttraumatic Stress Disorder (PTSD)<br>Suicidal ideation
Drought	12	Rural (8), Both (2)	Australia (4), Bangladesh, Botswana (2), Ethiopia, India (3), USA	Anxiety<br>Community drought-related stress<br>Depression (3)<br>Distress<br>Occupational psychosocial stress ("job strain")<br>Personal drought-related stress<br>Posttraumatic growth<br>Psychological distress (2)<br>Self-harm<br>Suicide deaths (2)<br>Suicide attempts<br>Suicide ideation (2)<br>Thoughts of self-harm<br>Trauma symptoms (2)
Flood	41	Rural (11)<br>Urban (14)<br>Both (4)<br>Semi-urban (1)<br>Suburban/ semi-rural (1)	Australia (2), Bangladesh (2), Burundi, Canada, China (7), Ghana, India (9), Malaysia (1), Indonesia (5), Pakistan, Spain, Thailand, UK (7), USA (2)	Anger<br>Anxiety (17)<br>Children's PTSD symptoms<br>Chronic PTSD<br>Chronic stress<br>Depression (22)<br>Depression, anxiety and stress<br>Drugs used in management of common mental disorders<br>Dysfunctional cognitions (2)<br>Emotional functioning – children<br>General psychiatric morbidity<br>Generalized Anxiety Disorder (GAD) (3)<br>Health related quality of life<br>Impact of event<br>Major Depressive Disorder (MDD)<br>Mania (2)<br>Mental health service utilization<br>Obsessive compulsive disorder<br>Panic disorder<br>Parent PTSD symptoms<br>Parent reported PTSD symptoms for children<br>Post Traumatic Stress Reactivity (3)<br>Posttraumatic cognition<br>Posttraumatic Stress Disorder (PTSD) (29)<br>Posttraumatic Stress Symptoms (PTSS) (2)<br>Probable anxiety<br>Probable depression<br>Probable PTSD<br>Problems with memory<br>Psychological distress<br>Psychosis (2)<br>Risk of suicide<br>Rumination<br>Schizophrenia admissions<br>Service use for all other mental health/behavioral disorders<br>Service use for anxiety disorders<br>Service use for bipolar disorders<br>Service use for depressive disorders<br>Service use for schizophrenic and psychotic disorders<br>Service use for substance and addictive disorders<br>Service use for trauma and stress disorders<br>Sleep disorders<br>Somatic symptoms

EWE	# of papers	Rural/urban (frequency)	Countries (frequency)	Mental Health Outcome Measures (frequency)
				Still distressed (2)<br>Stress (3)<br>Substance abuse (2)<br>Substance abuse – Alcohol<br>Suicidal attempts<br>Suicidal ideation (5)<br>Suicidal tendency<br>Suicide deaths<br>Symptoms of anxiety<br>Symptoms of depression<br>Symptoms of dissociation<br>Well-being
Extreme Cold/ cold spells	6	Urban (6)	China (4), Sweden, USA	Affective disorder outpatient visits (minus depression)<br>Anxiety outpatient visits<br>Depressive disorder outpatient visits<br>Mental disorder - admissions<br>Mental disorder outpatient visits<br>Mental health ED visits (2)<br>Mood disorder ED visits<br>Neurotic disorder ED visits<br>Psychiatric emergency visits<br>Psychoactive substance use ED visits<br>Schizophrenia admissions<br>Schizophrenia ED visits<br>Schizophrenia outpatient visits
Extreme heat / heat waves	32	Rural (1), Urban (18), Both (6)	Australia (3), Belgium, Brazil, Canada, China (7), Finland, India (2), Japan (3), Philippines, South Africa, South Korea (2), Spain (2), Sweden, Switzerland, Taiwan, UK USA (8), Vietnam (3)	Mental and behavioural disorders – admissions (3)<br>Adult personality and behavior disorder ED visits<br>Affective disorder outpatient visits (minus depression)<br>Alzheimer's deaths within 2 months of hospitalization<br>Alzheimer's disease urgent hospital admissions<br>Alzheimer's hospitalizations<br>Anxiety disorder ED visits<br>Anxiety emergency admissions<br>Anxiety outpatient visits<br>Anxiety, stress-related and somatoform disorder ED visits<br>Behavior and mood disorders usually associated with childhood and youth - admissions<br>Complex behavioural disorders - admissions<br>Complex behavioural disorders associated with physiological disorders and somatic factors admissions<br>Crisis help seeking<br>Dementia ED visits<br>Dementia emergency admissions<br>Depression emergency admissions<br>Depressive disorder outpatient visits<br>Depressive symptoms<br>Developmental disorders - admissions (3)<br>Mental and behavioral disorder related deaths<br>Mental and behavioral disorders - admissions<br>Mental and behavioral disorders caused by use of psychoactive substances - admissions (2)<br>Mental and behavioural disorder - ED visits<br>Mental disorder ED visits<br>Mental disorder outpatient visits<br>Mental disorders admissions (8)<br>Mental health admissions (2)<br>Mental health ED visits (6)<br>Mental health emergency admissions<br>Mental retardation admissions (2)<br>Mood (affective) disorders<br>Mood disorder ED visits (3)<br>Mood disorders - admissions (3)<br>Neurotic disorder ED visits<br>Neurotic disorder hospitalizations<br>Neurotic, stress-related and somatoform disorder admissions (3)<br>Psychiatric emergency visits<br>Psychoactive substance use ED visits (2)<br>Psychoactive substance use hospitalizations

EWE	# of papers	Rural/urban (frequency)	Countries (frequency)	Mental Health Outcome Measures (frequency)
				Schizophrenia - admissions (2)<br>Schizophrenia ED visits (2)<br>Schizophrenia emergency admissions<br>Schizophrenia outpatient visits<br>Schizophrenia, schizotypal and delusional disorders<br>Schizophrenia, schizotypal and delusional disorder ED visits<br>Self-harm ambulance cases<br>Self-harm ED visits<br>Substance use ED visits<br>Suicide deaths (6)<br>Suicide watch incidents<br>Suicide-related ambulance calls<br>Suicides per 100,000
Extreme rainfall	2	Urban (1), Both (1)	China (2)	Posttraumatic Stress Disorder (PTSD)<br>Resilience<br>Rumination<br>Schizophrenia admissions
Hurricane	21	Urban (10), Both (2), Suburban/ semi-rural (1)	Bahamas, Puerto Rico, USA (19)	Acute psychological distress<br>Adjustment disorder ED visits and hospitalizations<br>Adolescent psychological distress<br>Alcohol and substance use ED visits and hospitalizations<br>Alcohol use<br>Anxiety (3)<br>Anxiety related ED visits and hospitalization<br>Binge drinking in past year<br>Cannabis use<br>Cigarette use<br>Days a month of poor physical or mental health<br>Depression (5)<br>Depressive symptoms<br>Functional impairment<br>Generalized worries<br>Global distress<br>High Stress<br>Hurricane-related distress<br>Major depressive disorder (MDD) (2)<br>Mental health related ED visit and hospitalizations<br>Mood disorder ED visits and hospitalisations<br>Opioid abuse<br>Perceived stress<br>Poor mental health days<br>Posttraumatic stress<br>Posttraumatic Stress Symptoms (PTSS) (2)<br>Psychological distress<br>Psychosis ED visits and hospitalizations<br>Posttraumatic Stress Disorder (PTSD) (8)<br>Posttraumatic Stress Symptoms (PTSS) (2)<br>Recreational drug use<br>Sadness<br>Self-reported mental health difficulties in past 30 days<br>Substance abuse - alcohol (2)<br>Suicidal ideation (2)<br>Suicide and intentional harm ED visits and hospitalizations
Snowstorm	2	Urban (1), Both (1)	UK, USA	Emotional upheaval<br>Posttraumatic Stress Disorder (PTSD)
Tornado	6*	Urban (5), Both (1)	China (5), USA	Burnout (2)<br>Depression (2)<br>Mindfulness (3)<br>Parental Attachment<br>Posttraumatic Growth (PTG)<br>Posttraumatic Stress Disorder (PTSD) (4)<br>Posttraumatic Stress Symptoms (PTSS)<br>PTSD Reaction Index<br>Regulatory emotional self-efficacy
Typhoon	9	Urban (4), Both (4)	Philippines (8), Taiwan	Acute stress disorder (2)<br>Centrality of event and mental health outcomes<br>Depression

EWE	# of papers	Rural/urban (frequency)	Countries (frequency)	Mental Health Outcome Measures (frequency)
				Depression, anxiety, and stress (2)<br>Generalized Anxiety Disorder (GAD)<br>Insomnia<br>Monthly visits for adjustment reactions<br>Monthly visits for all stress-associated illness<br>Monthly visits for anxiety<br>Monthly visits for depressive disorders<br>Monthly visits for episodic mood disorders<br>Monthly visits for PTSD<br>Monthly visits for stress-associated insomnia<br>Positive metacognitions and positive meta-emotions<br>Posttraumatic cognitions (2)<br>Posttraumatic Growth (PTG)<br>Posttraumatic Stress Disorder (PTSD) (5)<br>Psychological distress (2)<br>Sensory-based memory quality of traumatic event
Wildfire	28	Rural (6), Urban (13), Both (3)	Australia (11), Canada (12), Greece, USA (4)	Agoraphobia<br>Alcohol use<br>Alcohol use disorder<br>Anger<br>Anxiety (6)<br>Avoidant attachment style<br>Coping strategies<br>Depression (10)<br>Drug use disorder<br>Drug-related problems<br>Fear of death<br>Fire-related PTSD<br>Generalized Anxiety Disorder (GAD) (5)<br>Grief<br>Insomnia (3)<br>Major Depressive Disorder (MDD) (6)<br>Major depressive episodes (2)<br>Mental and emotional well-being<br>Mental health service utilisation<br>Nicotine dependence<br>Obsessive-Compulsive Disorder<br>Panic disorder<br>Perceived stress<br>Perinatal PTSD<br>Peritraumatic dissociative experiences<br>Peritraumatic distress<br>Physical activity and well-being<br>Posttraumatic Growth (PTG)<br>Posttraumatic stress<br>Posttraumatic Stress Disorder (PTSD) (17)<br>Posttraumatic Stress Symptoms (PTSS)<br>Psychological distress (4)<br>Psychopathology symptoms<br>Psychotropic medication prescription rates - antidepressants<br>Psychotropic medication prescription rates - antipsychotics<br>Psychotropic medication prescription rates - anxiolytics<br>Psychotropic medication prescription rates - hypnotics<br>Psychotropic medication prescription rates - mood stabilizers<br>Severe mental illness<br>Social phobia<br>Somatic symptoms<br>Stress (2)<br>Substance abuse - Alcohol (5)<br>Substance abuse - Drugs<br>Substance abuse (3)<br>Suicidal ideation (2)<br>Tobacco use<br>Well-being

ED: Emergency Department; PTSD: Posttraumatic Stress Disorder

*5 of the 6 studies used the same baseline population data
